# Ethylene-driven enhancement of bioactive metabolites and *in vitro* functionality in soybean (*Glycine max* (L.) Merr.) and mung bean (*Vigna radiata* (L.) Wilczek) leaves grown in vertical farms: a comparative study

**DOI:** 10.1186/s12870-026-08829-8

**Published:** 2026-04-30

**Authors:** Mu Yeun Jang, Du Yong Cho, Hee Yul Lee, Jong Bin Jeong, Da Hyun Kim, Do Yun Bang, Hye Rim Kim, Ye Rim Jeong, Jin Hwan Lee, Kye Man Cho

**Affiliations:** 1 Department of GreenBio Science and Agri-Food Bio Convergence Institute, Gyeongsang National University, 139-8 Naedong-ro, Jinju, 52849 Republic of Korea; 2 Gyeongnam Anti-Aging Research Institute, Sancheong-gun, Gyeongsangnam-do 52215 Republic of Korea; 3 Department of Smart Green Resources, Dong-A University, 37 Nakdong-Daero 550 Beon-gil, Saha-gu, Busan, 49315 Republic of Korea; 4 Division of Food Science & Technology, Gyeongsang National University, 501 Jinju-daero, Jinju, 52828 Republic of Korea

**Keywords:** Soybean leaves, Mung bean leaves, Secondary metabolism, Hormonal regulation, Ethylene, Amino acids, Fatty acids, Isoflavones, DNA protection

## Abstract

**Background:**

Signal-induced augmentation of physiologically active metabolites in edible plants represents a promising approach for the development of high-value-added natural biological resources. Nevertheless, despite the potential of signaling molecules such as ethylene (ETL) to regulate plant metabolism, there are limited systematic studies comparing their organ-specific effects. Therefore, we conducted this comparative study to investigate the effects of ETL treatment on biomass production, accumulation of bioactive metabolites, and associated biological activities in soybean (Glycine max) and mung bean (Vigna radiata) leaves.

**Results:**

ETL exposure slightly reduced the plant height and significantly decreased the biomass in both species. Remarkably, ETL-treated mung bean leaves (ML) exhibited the highest total phenolic (24.22 mg GAE/g) and flavonoid (9.89 mg RE/g) contents, whereas soybean leaves (SL) demonstrated greater diversity and accumulation of amino acids such as γ-aminobutyric acid, leucine, and tyrosine. After ETL treatment, the total isoflavone contents increased markedly from 1440.43 to 8703.14 µg/g in SL (~6-folds) and from 2697.71 to 42,708.64 µg/g in ML (~16-folds), indicating a marked increase in isoflavonoid accumulation following ETL treatments. ETL treatment also improved the antioxidant capacity and digestive enzyme inhibition, with ML exhibiting greater radical scavenging activity and stronger inhibitory effects on lipase and α-glucosidase. DNA protection assays further indicated enhanced DNA protective effects against oxidative damage *in vitro* in both species. Overall, ETL-treated mung bean leaves showed the highest accumulation of secondary metabolites among the tested samples.

**Conclusions:**

Altogether, these results indicate that ETL elicits species-dependent differences in metabolite accumulation patterns and associated *in vitro* bioactivities, highlighting its potential as an elicitor-based approach for enhancing functional plant resources under controlled cultivation conditions.

**Graphical Abstract:**

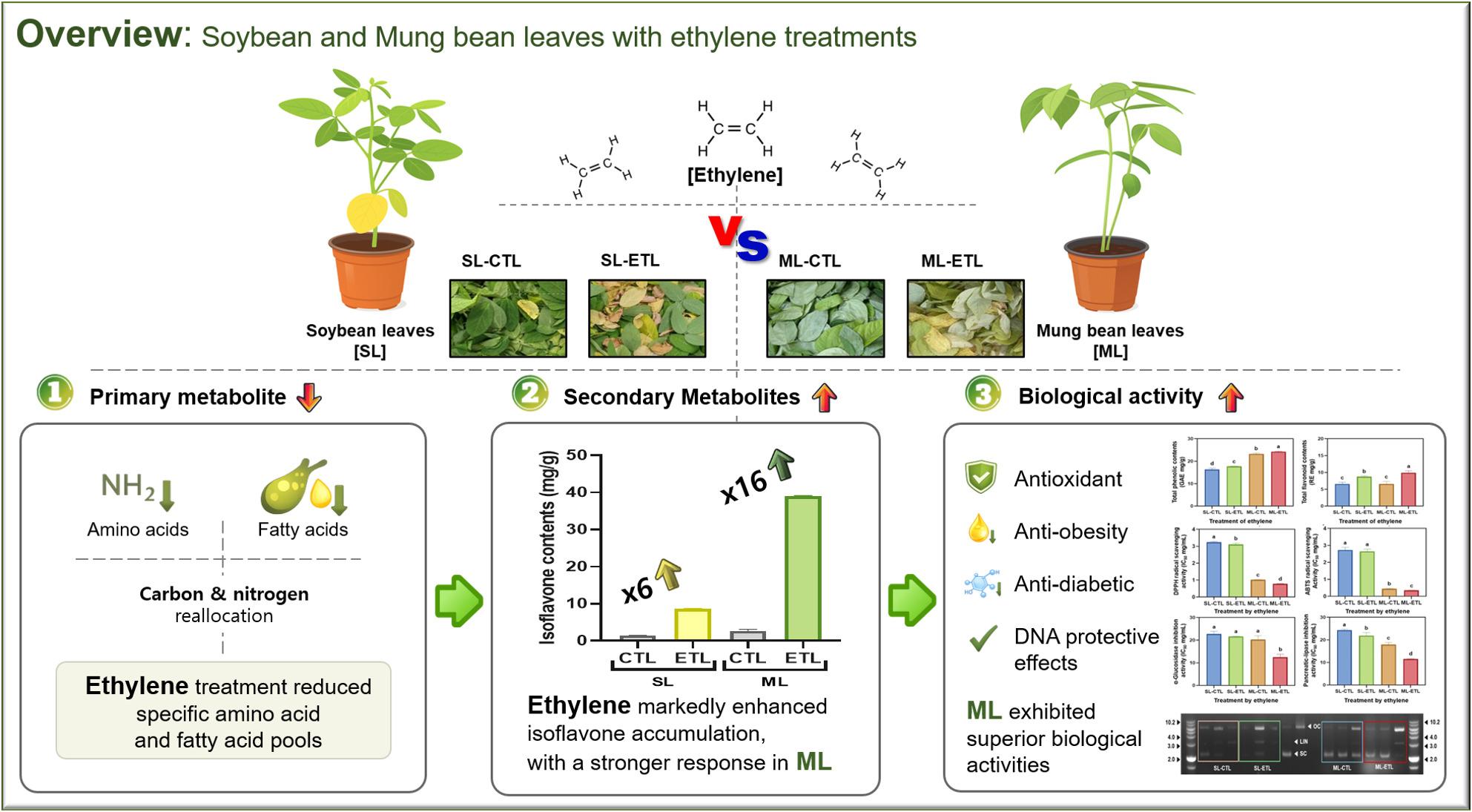

**Supplementary Information:**

The online version contains supplementary material available at 10.1186/s12870-026-08829-8.

## Highlights

ETL increased isoflavone content ~6-folds in SL and 16-folds in ML.

Change in FA and FAA profiles exhibited species-specific metabolic responses.

ML demonstrated higher antioxidant and enzyme inhibitory activities than SL.

ETL treatment improved DNA protection in both SL and ML.

**Supplementary Information:**

The online version contains supplementary material available at 10.1186/s12870-026-08829-8.

## Introduction

Metabolite farming has emerged as a promising strategy for enhancing the accumulation of target secondary metabolites in plants through controlled environmental and hormonal elicitation [1, 2]. By modulating intrinsic metabolic pathways in response to external stimuli, this approach enables the production of value-added plant materials with potential applications in functional foods, nutraceuticals, and bioresources [3, 4]. Secondary metabolites such as flavonoids and isoflavones play essential roles in plant defense and stress adaptation, and they have been widely investigated for their potential physiological relevance [5, 6].

Advances in controlled cultivation systems, including vertical farming and hydroponic platforms, now allow precise regulation of environmental factors that influence metabolite accumulation [1, 7].

Ethylene (ETL), a gaseous plant signaling molecule, influences diverse physiological processes, including senescence, stress responses, and specialized metabolism [8–10]. Previous studies have reported that exposure to plant hormones, such as methyl jasmonate, abscisic acid, and ETL, modulates secondary metabolite accumulation in various plant systems [3]. Previous studies have reported that ETL acts as a stress-associated regulator that can promote isoflavone biosynthesis in soybean by inducing reactive oxygen species (ROS) signaling and activating the phenylpropanoid pathway. In addition, exogenous ethephon treatment has been associated with ACS/ACO-mediated ETL biosynthesis and increased expression of structural genes such as *PAL*, *C4H*, *4CL*, *CHS*, and *IFS*, which are involved in flavonoid–isoflavonoid biosynthesis [11–13].

Despite recent advances in controlled elicitation strategies, a key question remains unresolved. Although soybean and mung bean share a common legume lineage, they differ in metabolic organization at both the primary and secondary metabolism levels. Variations in carbon–nitrogen coordination, amino acid turnover, and lipid metabolism (primary metabolism), as well as divergence in phenylpropanoid-derived isoflavone biosynthesis, conjugation patterns, and storage forms (secondary metabolism), may result in distinct metabolic reconfiguration under identical ethylene treatment conditions. However, whether closely related legume species exhibit differential metabolite accumulation patterns and functional responses under standardized ETL elicitation remains insufficiently characterized [1, 5, 11, 13]. Addressing this gap requires a systematic side-by-side comparison under controlled vertical farming environments.

Isoflavones are specialized metabolites belonging to the phytoestrogen family and are particularly abundant in legume species such as soybean leaves (SL) and mung bean leaves (ML) [5, 6]. Compounds including daidzein (DAE) and genistein (GEE) have been widely investigated for their potential physiological relevance [5, 14, 15]. In soybeans, these compounds occur in multiple conjugated forms, including aglycones and glycosides, and are distributed across vegetative tissues such as leaves and roots [16, 17]. Previous studies have reported that ethylene exposure is associated with increased isoflavone accumulation in soybean leaves [11, 12], while enhanced production of distinct isoflavone derivatives has been observed in ethylene-treated mung bean plants cultivated under vertical farming conditions [1].

However, systematic side-by-side comparisons of ethylene-associated changes in isoflavone composition and related metabolite classes between SL and ML under identical controlled cultivation environments remain limited.

Therefore, although previous studies reported increases in ETL-mediated metabolites in SL [11, 12] and ML [1], direct comparison of metabolite and functional responses under the same control conditions has not been systematically performed. In this study, we aimed to compare and evaluate *in vitro* functional indicators associated with metabolite accumulation patterns in SL and ML cultured and exposed to controlled ETL exposure under standardized vertical farming conditions. We hypothesized that ETL exposure would induce species-dependent metabolic responses between SL and ML, resulting in distinct metabolite accumulation patterns and associated biofunctional activities under identical cultivation conditions. To address this objective, we comparatively analyzed changes in primary metabolites, secondary metabolites, and their associated *in vitro* bioactivities under controlled ETL exposure. Through this comparative framework, we sought to clarify ETL-related metabolic responses of SL and ML in a controlled culture environment.

## Materials and methods

### Chemicals, reagents, and instruments

The reagents used for the *in vitro* determination of biological activity and total phenol (TP) and total flavonoid (TF) contents were purchased from Sigma-Aldrich (St. Louis, MO, USA). H_2_O, MeOH, EtOH, and acetonitrile (ACN) purchased from J.T. Baker (Phillipsburg, NJ, USA) were used as analysis and extraction solvents. For measuring the antioxidant activity, 2,2-diphenyl-1-pyridylhydrazine (DPPH) and 2,2′-azino-bis (3-ethylbenzothiazolin-6-sulfonic acid) (ABTS) radical scavenging were purchased from Sigma-Aldrich. (St, Louis, MO, USA).

The standard products used in this study were isoflavone and flavone derivatives such as daidzin (DAI), glycitin (GLI), genistin (GEI), DAE, glycitein (GLE), and GEE that were purchased from Sigma-Aldrich. Fatty acids (FA) composition was analyzed by gas chromatography (GC, Agilent 7890 A system, Agilent Technologies Inc., Wilmington, DE, USA). Free amino acids (FAA) composition was determined using an automatic amino acid analyzer (L-8900, Hitachi High-Technologies Corp., Tokyo, Japan). Isoflavones were analyzed by high-performance liquid chromatography (HPLC, Agilent 1260 series, Agilent Co., Forest Hill, Big, USA) to analyze. TP and TF analyses were performed using a spectrophotometer (Shimadzu UV-1800, Nakagyo-ku, Kyoto, Japan). *In vitro* biological activity was measured using an ELISA microplate reader (ELX800, Bio-Tek Instruments, Inc., Winooski, VT, USA).

DNA protection was evaluated using electrophoresis (Mupid-exU, Takara Bio Inc., Japan) and UV transilluminators (BioDoc-It™ 220 imaging system, Analytik Jena, Jena, Germany).

### Plant materials and growth conditions

The soybean seeds used in this study (Pungsan cultivar) were obtained from the Korea Agricultural Innovation Partnership (KOPIA, Jeonju, Republic of Korea) in 2021 and stored at 4 °C until use. Mung bean seeds were also obtained from KOPIA (Jeonju, Republic of Korea) in 2023 and stored at 4 °C before experimentation. The soybeans and mung beans were immersed in 2% sodium hypochlorite for 2 min and then washed with distilled water (DW). Sterilized soybeans and mung beans were immersed in DW and germinated at 25 °C for 24 ± 1 h in the dark. The sprouted soybeans and mung beans were then transplanted into pots with sterilized raised-bed soil (pH 4.0–7.0, EC 1.2 dS/m, Pungnong Co., Ltd., Korea). Then, 18 pots were placed in plastic containers (W × L × H, 540 × 275 × 60 mm). The plants were grown in a containerized vertical farm system under the following conditions: temperature 25 °C ± 2 °C, relative humidity 50% ± 10%, and light intensity 143.68 µmol·m^− 2^·s^− 1^ (light intensity 16 h; LED T5-1200 L-HC, Dongyoung Co., Ltd., Korea). The growth conditions were maintained for 28 days.

### ETL treatment conditions

The soybean and mung bean plants were subjected to slight modifications in a smart metabolite chamber (Supplementary Fig. 1) according to the method described by [1, 18]. After creating a smart metabolite chamber in a sealed environment before ETL treatment, temperature (25 °C ± 5 °C) and light intensity (143.20 µmol·m^− 2^·s^− 1^; photoperiod of 16 h) were maintained consistent with conditions in the vertical farm system, and humidity was maintained at 90% ± 5%. ETL (concentration 10,000 ppm) was applied onto soybean and mung bean plants twice (every 24 h) for 48 h [1, 18]. Finally, the soybean and mung bean plants were divided into a control (CTL) group (SL-CTL and ML-CTL) and an ETL-treated group (SL-ETL and ML-ETL) (Supplementary Fig. 1).

### Length and biomass measurement

The length and fresh and dry weights of CTL and ETL-treated soybean and mung bean plants after 28 days of sowing at the vertical farm were measured by random selection of 20 plants. Plant length was measured as the maximum length (cm) from the top of the cotyledon to the root end using a digital Vernier Caliper (Mitutoyo Kawasaki, Japan 552-304-10). Fresh weight was recorded using an electronic scale (HS2200S, Hanseong Equipment Co., Seoul, Korea) after separation of plant organs into leaves. Then, the plants were maintained at 55 °C in a dry oven (WOF-W155, DAIHAN Scientific Co., Ltd, Wonju, Korea) for 48 h before recording their dry weight. The GraphPad software (version 8.2.1, La Jolla, CA, USA) was used to determine significant differences in ETL treatment according to Tukey’s multiple test (*p* < 0.05), with the same row determined by analysis of variance (ANOVA).

### Sample extraction

SL and ML were harvested separately from five independent pots per treatment group (*n* = 5), weighed (fresh and dried weight), dried, and ground. For each biological replicate, 1 g of powdered sample was mixed with 20 mL of 50% MeOH in conical tubes as described by [1]. All samples were subjected to agitation extraction at 25 °C ± 2 °C for 24 h. The mixed extract was centrifuged at 3000 × *g* for 10 min, and the resulting supernatant was filtered using a 0.45-µm membrane filter (Whatman plc., Little Chalfont, Buckinghamshire, UK). HPLC, *in vitro* biological activity analysis, and DNA protection assay were performed using 50% MeOH extracts obtained from SL and ML.

### Measurement of FA contents

For FA contents analysis, sample preparation and pretreatment were performed using a method described by [18, 19] with modifications. Briefly, it was performed according to the FA esterification process. First, 50 mg of sample powder, 1 mL of 0.5 N NaOH dissolved in methanol, and 1 mL of triundecanoin (C_11:0_, 2 mg/mL), an internal standard, were mixed by vortexing. After heating the mixture at 100 °C for 5 min, 4 mL of 14% BF_3_ (Supelco, Bellefonte, PA, USA) was mixed and heated again at 100 °C for 30 min. Then, 2 mL of isooctane was added to the mixture and stirred vigorously for 30 s. Immediately, 10 mL of saturated NaCl solution was added and shaken. Next, the mixture was cooled to 25 °C, and the isooctane layer was separated from the aqueous solution and dehydrated with anhydrous sodium sulfite. The pretreated samples were filtered through a 0.45-µm membrane for GC analysis. Finally, the amount of FA in each sample was determined using an SP-2560 capillary column (100 m × 0.25 mm i.d., 0.2 μm film thickness, Supelco, St. Louis, MO, USA) and a GC column equipped with a flame ionization detector. The injection volume and injector temperature were set to 1 µL and 225 °C, respectively, and the system was operated in a split mode with a split ratio of 200:1. Helium was used as the carrier gas at a rate of 0.8 mL/min. The initial oven temperature was maintained at 100 °C for 4 min, then increased to 240 °C at a rate of 3 °C/min, and maintained at 240 °C for 15 min.

### Measurement of FAA contents

For FAA contents analysis, sample preparation and pretreatment were performed according to the amino acid analysis method, described previously by [18, 20]. Briefly, the program was processed according to the amino acid derivatization process. Treated samples were centrifuged at 15,000 × *g* for 3 min and filtered through a syringe filter. The filtrate was concentrated using a rotary evaporator (N-1300, Tokyo Rikakikai Co., Ltd., Tokyo, Japan) at 55 °C and then dissolved by adding 2 mL of lithium buffer (pH 2.2). This solution was filtered through a 0.45-µm membrane filter, and the final filtered sample was quantitatively analyzed using an automatic amino acid analyzer.

### Isoflavone analysis by HPLC

**Fig. 1:**
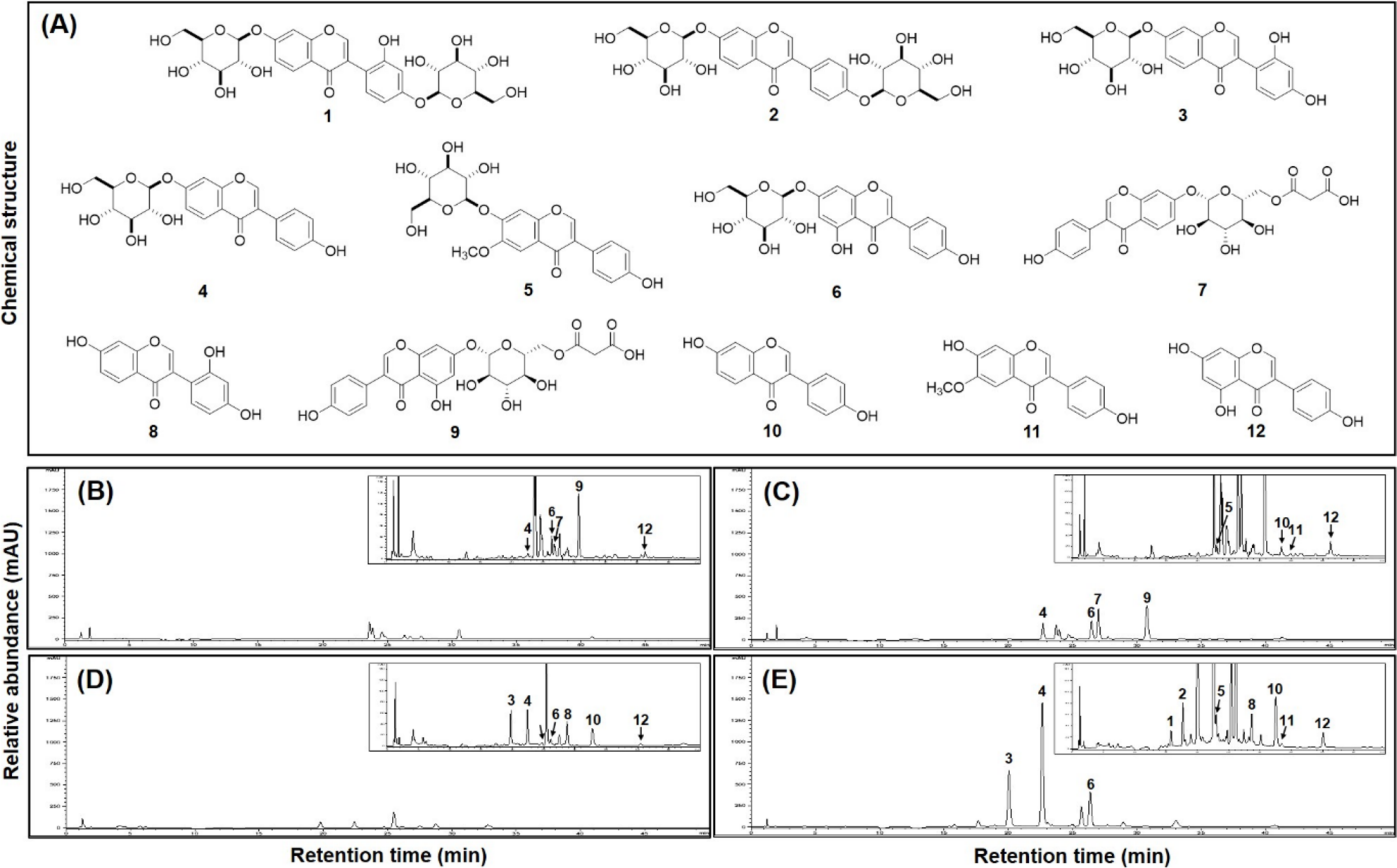
The chemical structures and typical HPLC chromatograms of 12 isoflavone derivatives. **A** Chemical structures of the identified isoflavone derivatives, including glycoside, malonyl-glycoside, and aglycone forms of daidzein, genistein, and glycitein, as well as their 2'-hydroxylated derivatives. **B** SL-CTL, control soybean leaves (untreated); **C** SL-ETL, ethylene-treated soybean leaves; **D** ML-CTL, control mung bean leaves (untreated); **E** ML-ETL, ethylene-treated mung bean leaves. 12 Chemicals: 1, 2'-hydroxydaidzein-4',7-O-diglucoside (2HDAEDG); 2, daidzein-4',7-O-diglucoside (DAEDG); 3, 2'-hydroxydaidzin (2HDAI); 4, daidzin (DAI); 5, glycitin (GLI); 6, genistin (GEI); 7, Malonyldaidzin (MDAI); 8, 2'-hydroxydaidzein (2HDAE); 9, Malonylgenistin (MGEI); 10, daidzein (DAE); 11, glycitein (GLE); 12, genistein (GEE)

The isoflavone contents of soybean and mung bean organ extracts were evaluated toward 12 standards (Fig. 1A). Isoflavone contents were determined using a LiChrospher 100 RP-18 column (4.6 × 250 mm, 5 μm). HPLC was performed using a diode array detector (1260 series, Agilent Co., Forest Hill, Big, USA) with the following settings: injection volume 20 µL, column temperature 30 °C, and absorbance 254 nm. The assay solvents were H_2_O containing 0.2% acetic acid (solvent A) and ACN containing 0.2% acetic acid (solvent B), and the gradient conditions were set to solvent B as follows: 15 min, 0%– 10%; 25 min, 10%–20%; 35 min, 20%–25%; 45 min, 25%–35%; and 50 min, 35% [1, 21]. The isoflavone contents of each sample were quantified by comparing the appropriate standard molecules and retention periods. The isoflavone standards were dissolved in DMSO and quantified using a linear calibration curve of each standard, and the coefficient of determination (R^2^) value was found to be > 0.998. Calibration curves were set at six points (6.25, 12.5, 25, 50, 100, and 200 µg/mL) for each standard.

### Measurement of TP and TF contents

The TP and TF contents were analyzed using the method described by [1, 21]. Briefly, the TP contents were first added to the extract (550 µL) in a test tube, followed by the addition of 25% Na_2_CO_3_ (550 µL), and left to stand for 3 min. Then, 300 µL of 2 N Folin–Ciocalteu phenol reagent was mixed with the sample and reacted for 1 h at 30 °C. Next, the optical density (OD) of the solution was measured using a spectrophotometer (Shimazu UV-1800, Nakachi, Kyoto, Japan) at 750 nm. Then, the TP contents were determined using a gallic acid (100, 50, 25, 12.5, and 6.25 µg/mL) standard curve. The TF contents were first mixed with 250 µL of sample extract and 500 µL of diethylene glycol *in vitro*. Then, 5 µL of 1 M NaOH was mixed with the sample and reacted for 1 h at 37 °C. Then, the OD of the solution was measured using a spectrophotometer at 420 nm. The TF contents were determined using a calibrated standard curve plotted using rutin (100, 50, 25, 12.5, and 6.25 µg/mL).

### *In vitro* biological activity analysis

The DPPH and ABTS radical scavenging activities and digestive enzyme inhibitory activities of CTL and ETL-SL and ML extracts were determined using previously described protocols [1, 21]. Briefly, the ABTS radical scavenging activity was determined by mixing 20 µL of extract with 180 µL of ABTS radical solution in a 96-well plate, incubating the plate at 30 °C for 3 min in the dark, and measuring the absorbance at 730 nm using an ELX800 microplate reader (Bio-Tek Instruments, Winooski, VT, USA). The DPPH radical scavenging activity was determined by mixing 40 µL of extract with 160 µL of 1.5 × 10^−^⁴ M DPPH in methanol, homogenizing, incubating the homogenate in the dark for 30 min at 30 °C, and measuring the absorbance at 515 nm. For determining the α-glucosidase inhibitory activity, 10 µL of extract was incubated with 20 µL of α-glucosidase (1 U/mL) and 20 µL of 0.01 M *p*-nitrophenyl-α-d-glucopyranoside for 20 min at 37 °C, after which the reaction was stopped by adding 150 µL of 0.1 M Na_2_CO_3,_ and the absorbance was measured at 420 nm. Pancreatic lipase inhibitory activity was analyzed by mixing 10 µL of extract with 20 µL of lipase (1 U/mL) and 20 µL of 0.01 M *p*-nitrophenyl butyrate, incubating the mixture for 20 min at 37 °C, terminating the reaction by adding 150 µL of 0.1 M Na_2_CO_3_, and then measuring the absorbance at 420 nm. All experiments were conducted using five independent biological replicates (*n* = 5). The half-maximal inhibitory concentrations (IC_50_) were calculated by nonlinear regression using GraphPad Prism v8.2.1 (La Jolla, CA, USA).

### Oxidative DNA damage protection analysis

The DNA-protecting effects of soybean and mung bean extracts from hydroxyl radicals were evaluated *in vitro* using supercoiled DNA (SC, pGEM-T Easy from *Escherichia coli*) as investigated previously [1, 16]. Plasmid DNA was extracted using 1 × TE buffer (10 mM Tris-HCl and 1 mM EDTA as elution buffer, pH 7.4) and Fenton’s reagent (100 mM H_2_O_2_, 0.1 mM acetic acid, and 1.6 mM FeCl_3_). First, 1 µL of pBluescript SK (+) vector, 5 µL each of soybean and mung bean sample extracts, and 4 µL of Fenton’s reagent were incubated at 30 °C for 1 h. Then, 25 mg of bromophenol, 25 mg of xylene cyanol, and 3 mL of 500 mM EDTA (pH 8.0) were mixed, and 50 mL of DW was used to prepare a 6 × DNA gel-loading dye. Then, 10 µL of the 6 × DNA gel-loading dye was mixed into the reaction samples, after which 10 µL of the dye mixture was loaded onto a 1.2% agarose gel. The gel was subjected to electrophoresis at 100 V for 40 min to observe the DNA and then photographed under a UV transilluminator. DNA retention (%) was calculated by comparing the band intensity of the pBluescript SK (+) plasmid in the presence of Fenton’s reagent and test samples with that of the untreated pBluescript SK (+) CTL using the following equation: DNA retention (%) = (band intensity of pBluescript SK (+) in Fenton’s reagent + sample/band intensity of untreated pBluescript SK (+) vector) × 100 (1).

### Statistical analysis and data processing

All experiments were conducted using five independent biological replicates (*n* = 5). Data are presented as mean ± standard deviation (SD). Significant differences among treatment groups were determined by one-way ANOVA using Statistical Analysis System (SAS) software (version 9.4, SAS Institute, Cary, NC, USA), followed by Tukey’s post hoc test at *p* < 0.05. Principal component analysis (PCA) and clustering heatmap analysis were performed using R version 4.3.3 (R Project for Statistical Computing, Vienna, Austria). The PCA plot, which combined the score plots, was normalized using the “prcomp” function and demonstrated using the “ggplot” package. The heatmap data were normalized using the Z-score, which is estimated using the following equation:

$$\:Z-\mathrm{s}\mathrm{c}\mathrm{o}\mathrm{r}\mathrm{e}\:=\:(\mathrm{x}\hspace{0.17em}-{\upmu\:}\hspace{0.17em})\:/\:{\upsigma\:}\:\left(2\right),$$

where x is the data value, µ is the mean, and σ is the standard deviation.

The clustering was visualized using Pearson correlation distance measure and Ward’s clustering method, which were performed using the “pHeatmap” package [1, 21]. PCA and clustering analyses were conducted using data obtained from five independent biological replicates per treatment group (*n* = 5). Clear separation among SL-CTL, SL-ETL, ML-CTL, and ML-ETL groups was consistently observed across biological replicates.

## Results

### Length and biomass of SL and ML groups under ETL treatment

**Fig. 2:**
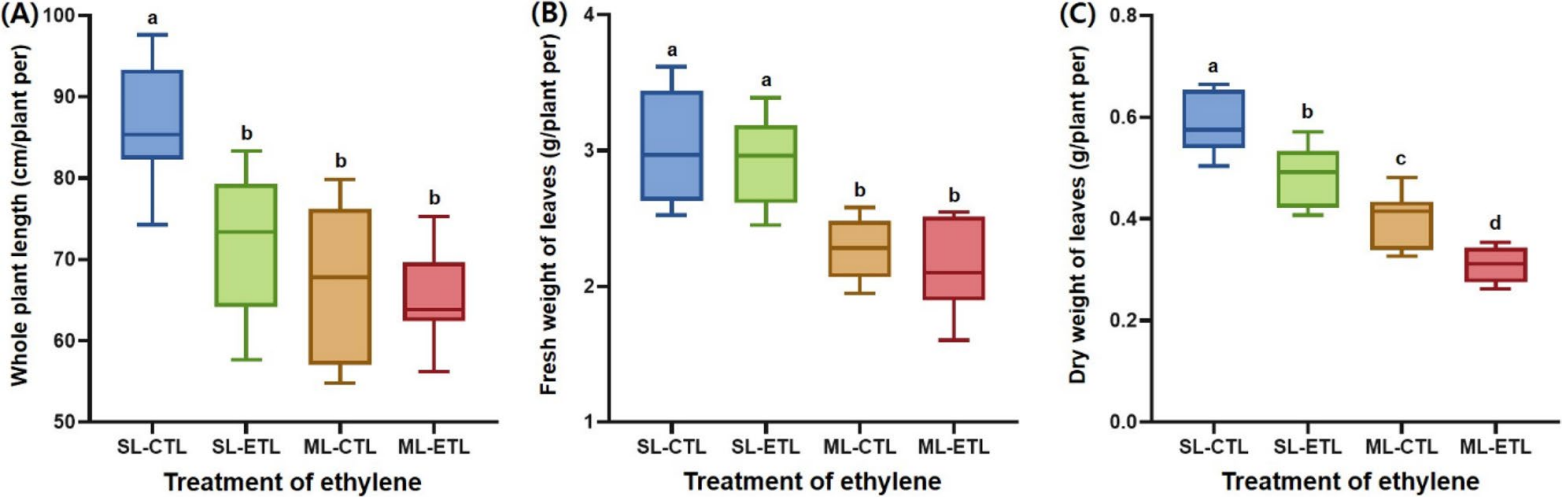
Effects of ETL treatment on plant length and leaf biomass in soybean and mung bean. **A** Whole-plant length, **B** leaf fresh weight, and (**C**) leaf dry weight under ETL treatment. Values are presented as mean ± SD (n = 5). Different lowercase letters indicate significant differences among treatments within each species, as determined by one-way ANOVA followed by Tukey’s multiple comparison test (p < 0.05). Abbreviation: SL-CTL, control soybean leaves (untreated); SL-ETL, ethylene-treated soybean leaves; ML-CTL, control mung bean leaves (untreated); ML-ETL, ethylene-treated mung bean leaves

Plant height differences between CTL and ETL groups are depicted in Fig. 1A. In soybean, the mean height changed from 86.70 to 71.67 cm, and in mung bean, it changed from 67.56 to 66.86 cm. Hence, ETL exerted a limited effect on plant height overall, and soybean plants remained taller than mung bean plants under both conditions. The impact of ETL exposure on biomass is shown in Fig. 2B and C. Regarding fresh weight (Fig. 2B), in soybean plants, it changed from 3.01 g in the SL-CTL group to 2.92 g in the SL-ETL group (0.970-fold); in mung bean plants, it changed from 2.26 g in the ML-CTL group to 2.04 g in the ML-ETL group. Therefore, ETL treatment reduced the fresh biomass in both species, with a larger proportional decrease in mung bean. Regarding dry weight (Fig. 2C), in soybean plants, it changed from 0.58 g in the SL-CTL group to 0.48 g in the SL-ETL group (0.828-fold); in mung bean plants, it changed from 0.40 g in the ML-CTL group to 0.31 g in the ML-ETL group. The reductions in dry mass were more prominent than those in fresh mass for both species. Across the CTL groups, both fresh and dry biomass of soybean exceeded those of mung bean, and this trend persisted even after ETL treatment. For each species, the ETL groups showed significantly lower biomass than their corresponding CTL groups (ANOVA/Tukey, *p* < 0.05).

These results indicate that ETL treatment caused statistically significant reductions in both fresh and dry biomass under the tested conditions, reflecting a clear growth penalty, particularly in mung bean. To distinguish true metabolic increases from potential concentration effects caused by biomass reduction, total isoflavone accumulation was recalculated per leaf. The results confirmed that ETL treatment significantly increased total isoflavone accumulation per leaf (Supplementary Fig. 2).

### Comparison of FA contents in soybean and mung bean plants after ETL treatment

**Fig. 3:**
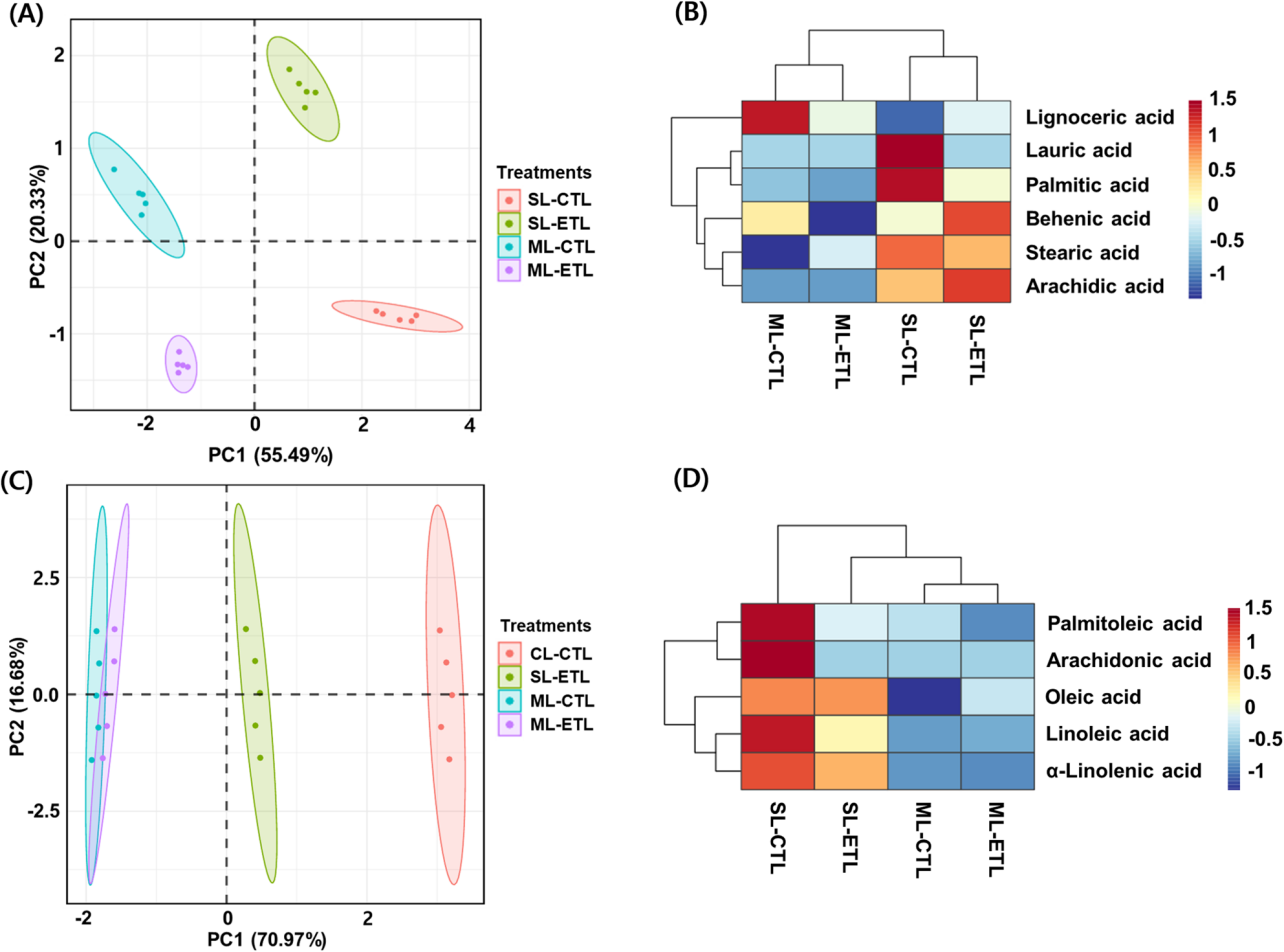
Visualization of the normalized data using Z-scores of leaf fatty acids in soybeans and mung beans through ethylene treatment. **A** a double plot with saturated fatty acid principal component analysis (PCA); **B** a heat map with saturated fatty acid Pearson correlation distance measurement and word clustering method; **C** a double plot with unsaturated fatty acid principal component analysis (PCA); and **D** a heat map with unsaturated fatty acid Pearson correlation distance measurement and word clustering method. The color scale represents the Z-score normalized abundance of metabolites, where red indicates higher accumulation and blue indicates lower accumulation relative to the mean

The comparison of FA profiles in SL and ML after ETL treatment is shown in Fig. 3 and Supplementary Table 1. In SL, the total saturated FA contents decreased from 391.9 to 365.9 mg/100 g after ETL exposure. Among individual saturated FA, palmitic acid content decreased from 273.7 to 244.7 mg/100 g, and stearic acid content decreased from 76.8 to 73.6 mg/100 g (0.958-fold) in SL. In ML, palmitic acid content remained almost unchanged, i.e., from 224.5 to 223.3 mg/100 g, whereas stearic acid content increased from 54.8 to 66.0 mg/100 g. Moreover, in SL, arachidic acid content increased significantly from 9.4 to 13.9 mg/100 g, behenic acid content increased significantly from 10.1 to 18.3 mg/100 g, and lignoceric acid content increased from 13.2 to 15.4 mg/100 g. However, in ML, arachidic acid and behenic acid contents decreased to less than the detection limit. Regarding unsaturated FAs, the total content in SL decreased from 816.6 to 629.3 mg/100 g, whereas in ML, it slightly decreased from 363.3 to 356.3 mg/100 g. In SL, palmitoleic acid contents decreased from 48.6 to 14.4 mg/100 g, linoleic acid content decreased from 215.5 to 127.1 mg/100 g, and α-linolenic acid content decreased from 495.6 to 440.5 mg/100 g, whereas arachidonic acid was undetectable. In ML, palmitoleic acid and arachidonic acid were also undetectable after ETL treatment, whereas oleic acid content increased from 27.5 to 37.2 mg/100 g, linoleic acid content increased from 61.4 to 66.6 mg/100 g, and α-linolenic acid content slightly decreased from 263.5 to 252.5 mg/100 g. The results of PCA further revealed that FA profiles in SL underwent substantial shifts after ETL treatment, with clear separation between the SL-CTL and SL-ETL groups along PC1 (55.49%) and PC2 (20.38%) axes. In contrast, the ML-CTL and ML-ETL groups exhibited minimal divergence, indicating limited metabolic response. These findings were corroborated by Z-score-based heatmap visualization, where SL exhibited considerable differences (decreased contents of palmitic/stearic acids and increased contents of arachidic/behenic/lignoceric acids). In contrast, ML showed no major changes, indicating that FA composition in response to ETL treatment was more altered in SL than in ML (Fig. 3B).

### Comparison of FAA contents in SL and ML after ETL treatment

**Fig. 4:**
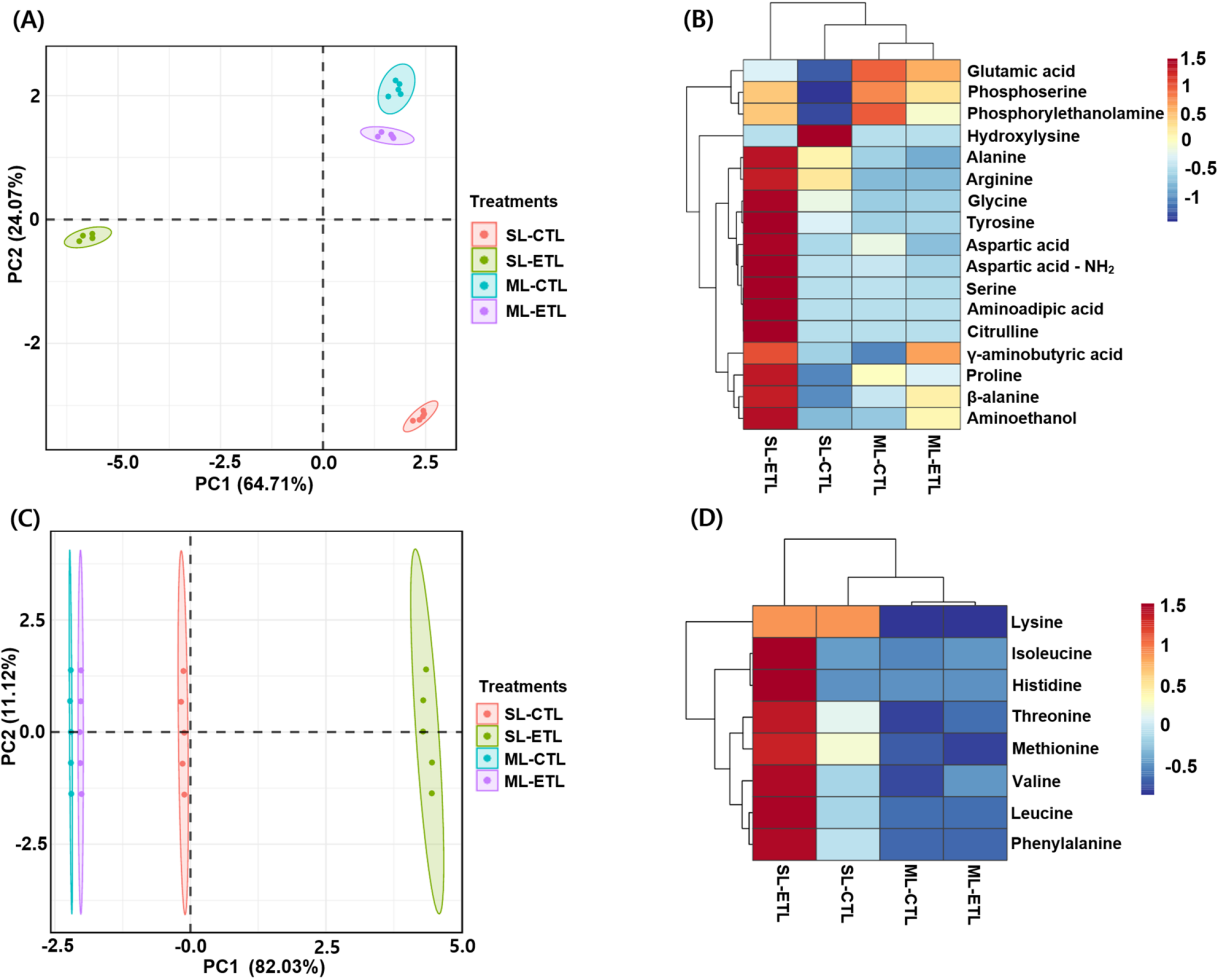
Visualization of the normalized data using leaf amino acid Z-scores from soybeans and mung beans through ethylene treatment. **A** a double graph with non-essential amino acid principal component analysis (PCA); **B** a heat map with non-essential amino acid Pearson correlation distance measurements and word clustering methods; **C** a double graph with essential amino acid fatty acid principal component analysis (PCA); and **D** a heat map with essential amino acid fatty acid Pearson correlation distance measurements and word clustering methods. The color scale represents the Z-score normalized abundance of metabolites, where red indicates higher accumulation and blue indicates lower accumulation relative to the mean

The comparison of FAA profiles in SL and ML under ETL treatment is depicted in Fig. 4 and Supplementary Table 2. In SL, ETL treatment induced a broad and coordinated elevation of both non-essential and essential amino acids. Among non-essential amino acids, the most pronounced increases were observed for aspartic acid-NH2 (8.05-fold), proline (5.37-fold), tyrosine (5.13-folds), and serine (3.10-fold). GABA (1.62-fold), glutamic acid (2.81-fold), and alanine (1.74-fold) also increased substantially. Among essential amino acids, isoleucine (5.83-fold), leucine (3.68-fold), phenylalanine (3.64-fold), and valine (2.15-fold) showed marked elevation. As a result, total FAA contents in SL increased from 446.07 to 1201.20 mg/100 g (2.69-fold) (Supplementary Table 2). PCA clearly separated SL-ETL from SL-CTL along PC1 (64.71% variance), driven primarily by the strong upregulation of aspartic acid-NH_2_, proline, tyrosine, isoleucine, and leucine (Fig. 4A). Heatmap visualization (Fig. 4B) further confirmed a broad enrichment pattern across most amino acids in SL-ETL. Together, these results indicate that ETL treatment triggered a systemic increase in nitrogen-associated metabolites in soybean leaves. In contrast, ML exhibited a more selective and limited response. Several amino acids, including aspartic acid-NH_2_, aspartic acid, glutamic acid, proline, and phosphoserine, decreased after ETL treatment. However, GABA (1.73-fold), β-alanine (1.42-fold), and valine (1.37-fold) increased. Consequently, total FAA contents in ML increased only modestly, from 397.05 to 431.17 mg/100 g (1.09-fold) (Supplementary Table 2). PCA did not clearly separate ML-ETL from ML-CTL along PC1 (82.03% variance), indicating a comparatively weaker metabolic shift (Fig. 4C). The heatmap (Fig. 4D) similarly reflected limited changes, with only GABA, β-alanine, and valine showing noticeable enrichment. Collectively, these findings reveal a species-dependent pattern in FAA remodeling under ETL treatment, characterized by broad nitrogen flux enhancement in soybean and selective stress-associated amino acid accumulation in mung bean.

### Comparison of isoflavone content in SL and ML under ETL treatment

**Fig. 5:**
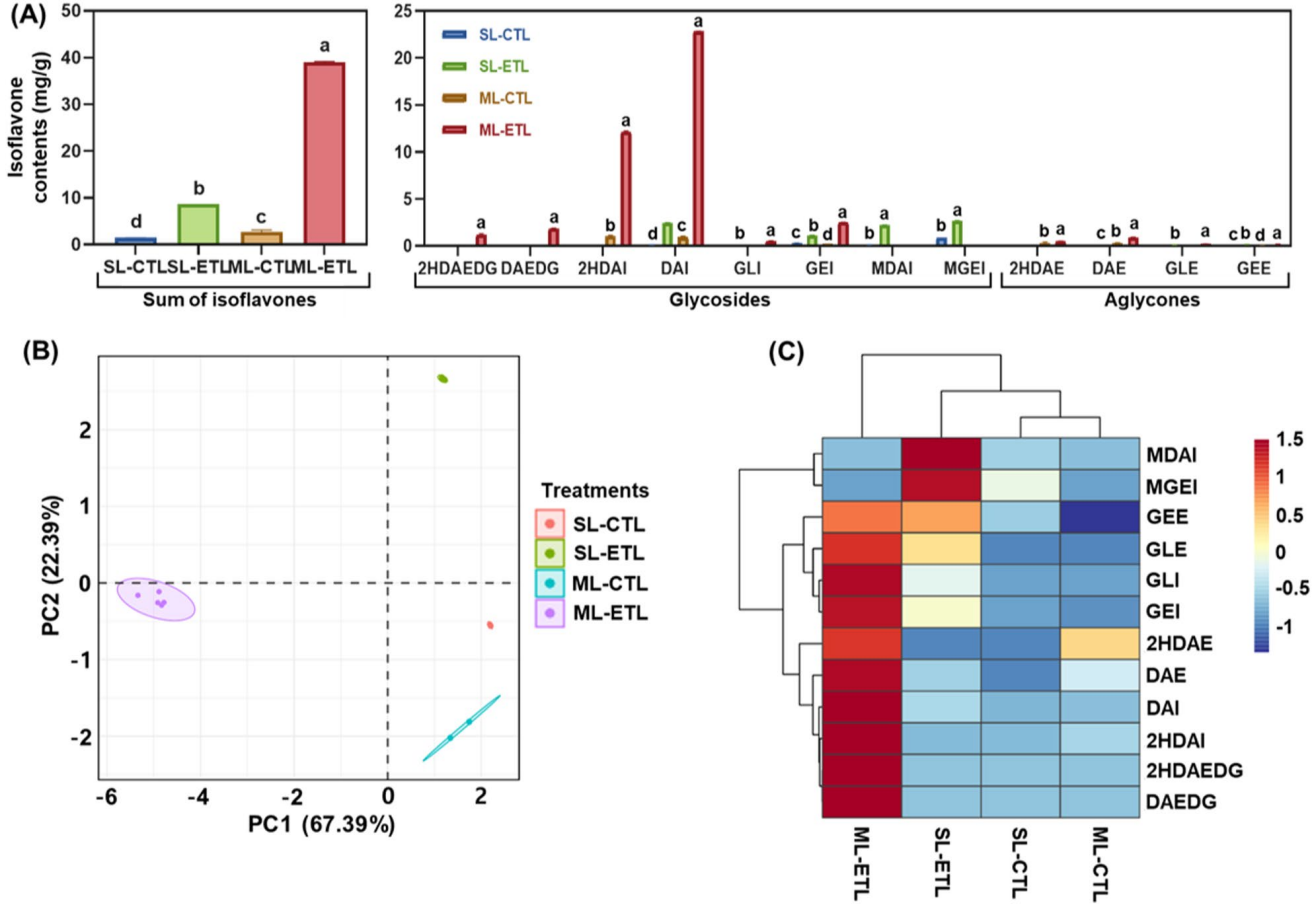
Visualization of the normalized data using the leaf isoflavone Z-scores of soybeans and mung beans through ethylene treatment. **A** isoflavone contents; **B** a double graph with isoflavone principal component analysis (PCA); **C** a heat map with isoflavone Pearson correlation distance measurements and word clustering methods. Values are presented as mean ± SD (n = 5). Different lowercase letters indicate significant differences among treatments within each species, as determined by one-way ANOVA followed by Tukey’s multiple comparison test (p < 0.05). The color scale represents the Z-score normalized abundance of metabolites, where red indicates higher accumulation and blue indicates lower accumulation relative to the mean

ETL treatment increased the total isoflavone content in both species, with a larger absolute increase in ML than in SL (Fig. 5A; Supplementary Table 3). The total isoflavone content increased 6.04-folds in SL (from 1440.43 to 8703.14 µg/g) and 15.83-folds in ML (from 2697.71 to 42,708.64 µg/g). As shown in Supplementary Table 3, the malonyl conjugates malonyldaidzin (MDAI) and malonylgenistin (MGEI) accumulated strongly in SL after ETL treatment, whereas they were not detected in ML. PCA clearly separated ETL-treated samples from controls in both species (Fig. 5B). PC1 (67.39%) captured the gradient in total isoflavones and major glycoside classes. The ML-ETL group was primarily driven by high loadings of glycosides such as DAI, 2′-hydroxydaidzin (2HDAI), GEI drove ML-ETL, whereas malonyl derivatives (MDAI and MGEI) characterized SL-ETL. The ML-ETL group showed extremely high levels of DAI, 2HDAI, GEI, as well as the diglucoside forms 2ʹ-hydroxydaidzein-4ʹ,7-*O*-diglucoside (2HDAEDG), and daidzein-4ʹ,7-*O*-diglucoside (DAEDG). Consistently, the SL-CTL and ML-CTL groups clustered on the negative side of PC1, reflecting low overall isoflavone levels. Heatmap visualization further supported this species-dependent metabolic signature (Fig. 5C), showing dominant glycoside accumulation in ML-ETL and prominent malonyl enrichment in SL-ETL. Compound identities were first established by HPLC-DAD using authentic standards and diagnostic UV spectra (Fig. 1B–E) and 12 isoflavone derivatives were quantified. At the compound levels, ETL significantly increased core glycosides (DAI/GLI/GEI) and malonyl forms (MDAI/MGEI) in SL (ANOVA, *p* < 0.05). In ML, the response was larger in magnitude, with dominance of glycosides and 2′-hydroxy-glycosides, including DAI (22,828.21 µg/g), 2HDAI (10,059.58 µg/g), GLI (3532.68 µg/g), and GEI (2502.84 µg/g). Diglycosides (2HDAEDG and DAEDG) were detected in ML but not in SL, whereas malonyl conjugates were absent in ML. Together, these results highlight distinct species-dependent modification patterns, with soybean preferentially accumulating malonyl conjugates and mung bean exhibiting strong glycoside and 2′-hydroxy-glycoside enrichment under ETL treatment.

### Comparison of TP and TF contents and *in vitro* biological activity

Regarding the TP and TF contents of SL and ML before and after ETL treatment, the ML-ETL group exhibited the highest levels of TP at 24.22 mg GAE/g and TF at 9.89 mg RE/g (Table 1). The ML-CTL group also exhibited significantly higher TP (23.24 mg GAE/g) and TF (6.60 mg RE/g) contents than both the SL-CTL and SL-ETL groups. In SL, the TF content increased from 6.57 mg RE/g in the SL-CTL group to 8.74 mg RE/g in the SL-ETL group, and the TP content also increased slightly from 16.31 to 17.71 mg GAE/g. Although the increase in TP content between the SL-CTL and SL-ETL groups was relatively small, TF content increased significantly after ETL treatment. The radical-scavenging activity was measured by comparing the IC50 values (mg/mL) for DPPH and ABTS (Table 1). For DPPH radical scavenging activities, the IC_50_ value of the SL-CTL group was 3.24 mg/mL, which slightly improved to 3.11 mg/mL in the SL-ETL group. However, in ML, the IC_50_ value decreased markedly from 1.02 mg/mL in the ML-CTL group to 0.78 mg/mL in the ML-ETL group, indicating an improvement of ~ 23.5% after ETL treatment. The DPPH radical scavenging activities IC_50_ value of the SL-ETL group (3.11 mg/mL) was only slightly lower than that of the SL-CTL group (3.24 mg/mL), whereas it was substantially lower in the ML-ETL group (0.78 mg/mL) than in the ML-CTL group (1.02 mg/mL), clearly demonstrating that ETL treatment strongly improved the DPPH radical scavenging capacity in ML. A similar trend was detected for the ABTS radical scavenging activity. No significant difference was observed in the ABTS radical scavenging activities IC_50_ value between the SL-CTL (2.73 mg/mL) and SL-ETL (2.65 mg/mL) groups; however, in ML, it decreased from 0.44 mg/mL in the ML-CTL group to 0.35 mg/mL in the ML-ETL group, indicating an improvement after ETL treatment. Remarkably, among all experimental groups, the ML-ETL group exhibited the lowest IC_50_ value, confirming that ETL-treated ML possess superior radical scavenging activity than SL. The *in vitro* biological activity of digestive enzymes was also evaluated by comparing the IC_50_ values for α-glucosidase and pancreatic lipase inhibitory activities. For α-glucosidase, the IC_50_ value decreased slightly from 22.73 mg/mL in the SL-CTL group to 21.64 mg/mL in the SL-ETL group, indicating a minor improvement (Table 1). However, in ML, the IC_50_ value decreased significantly from 20.22 mg/mL in the ML-CTL group to 12.49 mg/mL in the ML-ETL group, showing a reduction of ~ 38.2%, which emphasizes a much stronger improvement of α-glucosidase inhibition after ETL treatment. A comparison of these data indicates that the change in SL was marginal, whereas the improvement in ML was remarkable. Similarly, the IC_50_ value of pancreatic lipase inhibitory activity decreased by ~ 10.1% in SL (SL-CTL: 24.31 mg/mL and SL-ETL: 21.86 mg/mL), whereas in ML, it decreased by ~ 35.6% (ML-CTL: 17.90 mg/mL and ML-ETL: 11.53 mg/mL). This result indicates that the *in vitro* biological activity significantly improved in ML after ETL treatment. To further elucidate the relationship between metabolite accumulation and functional outcomes, Pearson correlation analysis was performed between total isoflavones, TP, and TF and bioactivity indices (DPPH, ABTS, α-glucosidase inhibition, and lipase inhibition). The analysis revealed significant correlations between isoflavone and phenolic contents and antioxidant and enzyme inhibitory activities (*p* < 0.05), supporting a quantitative association between metabolite enrichment and functional improvement (Supplementary Fig. 3).

**Table 1:** Comparison of total phenolic and flavonoid contents and radical scavenging and digestive enzyme inhibitory activities of soybean and mung bean leaves by treatment with ethylene

Index^1)^	Treatment of ethylene^2)^			
	SL-CTL	SL-ETL	ML-CTL	ML-ETL
Total phenolic contents (GAE mg/g)	16.31 ± 0.47^d^	17.71 ± 0.06^c^	23.24 ± 0.31^b^	24.22 ± 0.22^a^
Total flavonoid contents (RE mg/g)	6.57 ± 0.45^c^	8.74 ± 0.19^b^	6.60 ± 0.61^c^	9.89 ± 0.50^a^
DPPH radical scavenging IC_50_ (mg/mL)	3.24 ± 0.04^a^	3.11 ± 0.07^b^	1.02 ± 0.02^c^	0.78 ± 0.01^d^
ABTS radical scavenging IC_50_ (mg/mL)	2.73 ± 0.13^a^	2.65 ± 0.11^a^	0.44 ± 0.02^b^	0.35 ± 0.00^c^
α-glucosidase inhibition IC_50_ (mg/mL)	22.73 ± 0.97^a^	21.64 ± 0.16^a^	20.22 ± 1.44^a^	12.49 ± 1.05^b^
Pancreatic-lipase inhibition IC_50_ (mg/mL)	24.31 ± 0.33^a^	21.86 ± 1.11^b^	17.90 ± 0.77^c^	11.53 ± 0.16^d^

*Abbreviations*: *SL-CTL* control soybean leaves (untreated), *SL-ETL* ethylene-treated soybean leaves, *ML-CTL* control mung bean leaves (untreated), *ML-ETL *ethylene-treated mung bean leaves

^1)^All values are expressed as the mean ± SD of pentaplicate determination. Different small letters (a–d) correspond to significant differences related to the same row, as determined by the ANOVA and followed by Tukey’s multiple tests (*p* < 0.05)

^2)^Treatment conditions in plant chamber: light intensity 143.20 µmol/m^− 2^/s^− 1^ (16 h photoperiod), temperature 25 °C ± 5, humidity 90%±5, and ethylene concentration 10,000 ppm applied for 24 h, repeated twice (total exposure time: 48 h)

### *In vitro* plasmid DNA protection assay

**Fig. 6:**
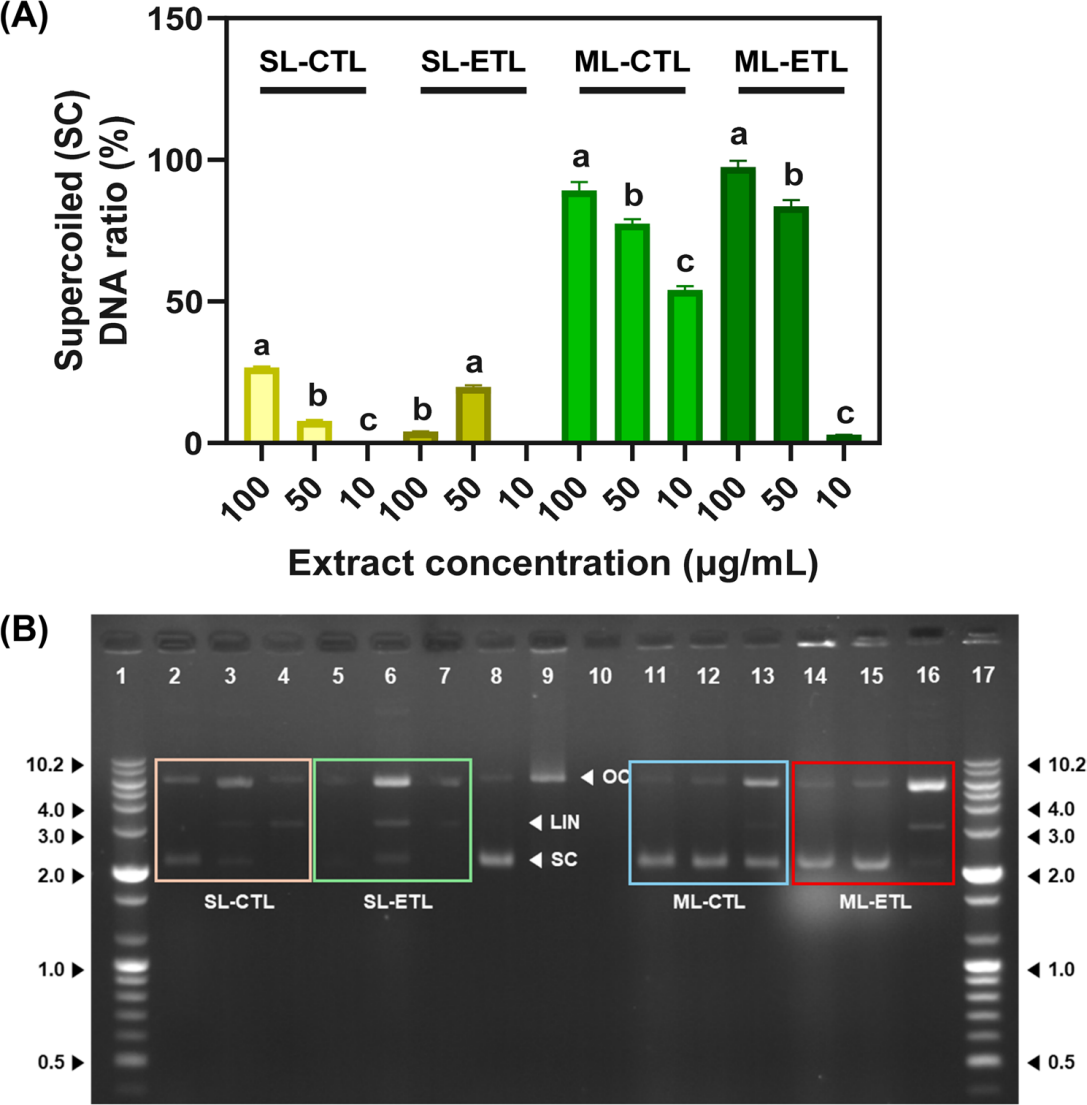
*In vitro* plasmid DNA protection assay of soybean and mung bean leaf extracts under ethylene treatment. **A** Quantitative analysis of supercoiled (SC) DNA percentage based on band intensity measured using ImageJ software. The SC DNA ratio (%) was used as an indicator of protection against oxidative strand breakage. Values are presented as mean ± SD (n = 5). Different small letters indicate significant differences among treatments at the same concentration (ANOVA followed by Tukey’s test, p < 0.05). **B** Representative agarose gel electrophoresis images showing the protective effects of leaf extracts against Fenton’s reagent-induced oxidative DNA damage. Lane 1 and 17, size marker; lanes 2–4, SL-CTL (100, 50, 10 µg/mL); lanes 5–7, SL-ETL (100, 50, 10 µg/mL); lane 8, pBluescript SK(+); lane 9, pBluescript SK(+) + Fenton’s reagent (H₂O₂); lane 10, 50% MeOH control; lanes 11–13, ML-CTL (100, 50, 10 µg/mL); lanes 14–16, ML-ETL (100, 50, 10 µg/mL). Abbreviations: SC, supercoiled DNA; OC, open circular DNA; LIN, linear DNA

We evaluated the *in vitro* plasmid DNA protection capacity of SL and ML extracts under oxidative stress conditions, and the proportion of the supercoiled DNA (SC) band was quantified as an indicator of oxidative damage protection (Fig. 6A, B). All values represent the mean ± SD of five independent experiments. In SL, the SC ratio in the SL-CTL group decreased in a concentration-dependent manner (26.84%, 7.85%, and 0.35% at 100, 50, and 10 µg/mL, respectively). In contrast, SL-ETL showed a higher SC retention at 50 µg/mL (19.73%) compared with SL-CTL (7.85%), whereas the SC ratios at 100 µg/mL (4.19%) and 10 µg/mL (0%) were not consistently elevated relative to the control. Overall, the DNA-protective effect in soybean was moderate and concentration-dependent. In ML, markedly higher SC retention was observed compared with soybean. In the ML-CTL group, SC ratios were 90.13%, 76.95%, and 53.40% at 100, 50, and 10 µg/mL, respectively. ML-ETL exhibited further enhancement at 100 µg/mL (99.32%) and maintained high protection at 50 µg/mL (82.30%), although SC retention declined sharply at 10 µg/mL (2.82%). Direct comparison between species indicated that mung bean extracts, particularly under ETL treatment at 100 µg/mL, exhibited the highest SC retention among all groups. These results demonstrate that ETL treatment enhanced *in vitro* DNA protection capacity more prominently in ML than in SL under the tested conditions.

## Discussion

Although ETL treatment slightly reduced plant height in both soybean and mung bean, statistically significant reductions were observed in fresh and dry biomass, particularly in leaf tissues. ETL has been implicated in the regulation of plant growth and senescence-related processes, with reported effects on chlorophyll metabolism and photosynthetic parameters depending on species and environmental conditions [22, 23]. Under stress-like hormonal signaling, carbon and metabolic resources may be redistributed from growth-associated processes toward defense-related secondary metabolism [24]. Thus, the biomass reduction observed in this study may be associated with ethylene-mediated modulation of photosynthetic performance and senescence-related processes. However, because photosynthetic parameters or chlorophyll-related markers were not directly measured, this interpretation remains inferential. In addition, ETL-treated samples exhibited noticeable changes in leaf coloration compared with the control (Supplementary Fig. 1) [12]. These visual changes may be associated with chlorophyll degradation and senescence-related responses induced by ETL [25]. However, since photosynthetic pigments were not directly quantified in this study, further investigations are required to confirm this relationship. Importantly, the reduction in biomass did not coincide with diminished functional quality. Instead, ETL treatment markedly enhanced the accumulation of bioactive metabolites, including isoflavones, and significantly increased TP and TF contents, particularly in mung bean leaves (Fig. 5; Table 1). Previous studies have shown that ethylene or its precursor ACC can upregulate key phenylpropanoid pathway genes, such as *PAL* and *CHS*, thereby stimulating flavonoid biosynthesis under specific stress-related conditions [26, 27]. Accordingly, the enhanced TP, TF, and isoflavone levels observed here are consistent with previously reported activation of phenylpropanoid-derived secondary metabolism under ethylene signaling. The concurrent increase in isoflavones and FAAs, despite reduced biomass, suggests that ETL treatment altered the metabolic profile in a manner that favored the enrichment of specific secondary metabolites under controlled cultivation conditions [18]. Although biomass was significantly reduced, the fold increase in isoflavone accumulation was substantially greater, suggesting that functional gain per unit biomass may compensate for yield loss in quality-oriented production systems. Nevertheless, because metabolite levels were expressed on a dry-weight basis, part of the apparent increase may reflect a concentration effect resulting from biomass reduction, in addition to genuine metabolic regulation. To address this possibility, total isoflavone accumulation was recalculated on a per-leaf biomass basis (Supplementary Fig. 2). The results confirmed that ETL treatment still resulted in higher total isoflavone accumulation per leaf, particularly in ML, indicating that the observed increases were primarily due to enhanced metabolic biosynthesis rather than to simple concentration effects caused by reduced biomass [1, 18]. This pattern aligns with the concept of metabolite farming, in which environmental or hormonal modulation is applied to enhance phytochemical concentration rather than maximize biomass yield [1].

However, as only a single ETL regime was evaluated, further studies examining dose- and time-dependent responses will be necessary to determine the optimal balance between biomass production and phytochemical enrichment. Moreover, the species-specific sensitivity to ETL, reflected by the greater biomass reduction in soybean compared with mung bean, highlights the importance of crop-dependent physiological responsiveness when applying hormonal elicitation strategies [28].

In the present study, ETL treatment induced species-dependent shifts in FA composition rather than broad changes in total lipid content. SL exhibited reductions in total unsaturated FAs accompanied by relative increases in very-long-chain saturated FAs, whereas ML maintained comparatively stable unsaturated FA levels (Fig. 3). Previous studies in Arabidopsis leaves have reported that ethylene signaling interacts with lipid regulatory networks and can influence FA composition under stress-related conditions [29]. These findings suggest that ETL-responsive signaling may modulate membrane-associated lipid organization as part of adaptive responses. Our observations in soybean are consistent with hormone-associated compositional adjustment patterns reported in ethephon-treated SL [18]. However, responses to plant hormones such as ETL and methyl jasmonate can vary considerably depending on plant species and treatment conditions, as shown in previous crop-based studies [30, 31]. The relative stability observed in the mung bean further supports the notion that ETL-associated lipid responses are species-dependent. Mung bean has been reported to contain high proportions of essential unsaturated FAs, including linoleic and α-linolenic acids [32], and maintenance of these components under ETL exposure may reflect tighter membrane lipid homeostasis.

Therefore, FA compositional shifts likely represent secondary physiological adjustments rather than primary determinants of functional enhancement [33].

FAAs are the basic building blocks of proteins and peptides, and act as precursors for various physiological pathways that maintain plant growth and defense [18]. In particular, phenylalanine is a key substrate of the phenylpropanoid pathway and is an important mediator linking primary nitrogen metabolism and the biosynthesis of secondary metabolites, including isoflavones [34]. GABA also plays a crucial role in plant growth and stress adaptation, and its accumulation is closely related to antioxidant capacity [35, 36]. In this study, ETL treatment significantly increased the total free amino acid content in SL, whereas relatively limited changes were observed in ML. These differences suggest species-specific nitrogen metabolic responses to ETL treatment [37, 38]. In particular, significant increases in aspartic acid-NH2 (asparagine), proline, tyrosine, and several branched-chain amino acids were observed in SL [39]. Asparagine serves as the main nitrogen storage and transport form in plants and is synthesized by asparagine synthetase. The expression of asparagine synthetase is regulated by specific transcription factors and has been reported to be modulated by stress and hormonal signals [40]. Previous studies have reported that ETL or its analog ethephon treatment can regulate the expression of nitrogen assimilation-related enzymes such as asparagine synthetase and glutamine synthetase, while simultaneously inhibiting some amino acid degradation pathways [18, 40, 41]. These prior studies support the possibility that the increase in asparagine and related amino acids observed in SL in this study was due to the regulation of nitrogen assimilation pathways by ETL. Furthermore, there is a report that ETL-based growth regulator treatment increases both essential and nonessential amino acids [42], suggesting that ETL may influence amino acid synthesis and turnover beyond a simple signaling factor. In contrast, in ML, a selective increase in specific amino acids such as GABA, β-alanine, and valine was observed, while the overall amino acid pool remained relatively stable. This suggests selective regulation of metabolic pathways associated with stress mitigation rather than changes in the broader nitrogen pool [43, 44]. These different response patterns may reflect differences in nitrogen metabolism regulation among species in response to ETL treatment. However, since this study did not analyze the expression of nitrogen assimilation enzymes or transcription factor levels, future transcriptome and enzyme activity analyses are needed to more clearly elucidate the effects of ETL on asparagine biosynthesis and nitrogen distribution pathways.

Furthermore, correlation analysis between metabolite classes revealed significant associations between isoflavone derivatives and several amino acids and fatty acids (Supplementary Fig. 4), suggesting coordinated metabolic regulation between primary and secondary metabolism under ETL treatment. The coordinated changes among isoflavones, phenolic compounds, and antioxidant activity suggest an integrated metabolic response associated with enhanced secondary metabolite production under ETL treatment, potentially linked to the phenylpropanoid pathway and its interaction with primary nitrogen metabolism [45].

Isoflavones are secondary metabolites belonging to the flavonoid family and are abundant in legumes. They play an important role in plant stress responses through antioxidant and cell protection activities [46]. Previous studies have reported that ETL or ethephon treatment may influence the expression of key structural genes involved in isoflavonoid biosynthesis [1, 12, 18]. These genes include *CHS*, *IFS*, and chalcone isomerase (*CHI*), which have been reported to be associated with isoflavone biosynthesis in legumes [12, 47]. Previous studies have reported induction of *IFS* and related enzymes after ETL treatment, suggesting that ETL may be involved in the regulation of the phenylpropanoid–flavonoid–isoflavonoid pathway in soybean systems. However, since we did not perform gene expression assays in the present study, the observed increase in isoflavone content could not be directly attributed to the transcriptional activation of these enzymes. In our experiments, ETL treatment significantly increased the total isoflavone content in both SL and ML. Since isoflavone concentrations were calculated on a dry weight basis and ETL treatment significantly reduced biomass in both species, we cannot rule out the possibility that some of the observed increases reflect the concentration effects associated with tissue mass reduction [18, 48]. Remarkably, ML has a 30-fold increase in absolute levels of DAI compared to CTL, which is consistent with previous findings [1]. The increased isoflavone accumulation is closely associated with improved protection against oxidative DNA damage [1], and it has been reported that biotransformation processes enhance the levels of physiologically active isoflavone forms and antioxidant potential in soybean systems [49]. Isoflavones contribute to DNA stabilization primarily by eliminating ROS and chelating antioxidant metal ions. In addition, glycoside isoflavones have higher solubility, improved intracellular mobility, and higher antioxidant stability compared to the aglycon forms, which may provide functional benefits [18]. The markedly larger isoflavone amplification observed after ETL treatment may also reflect species-specific differences in flavonoid biosynthesis regulation. Species-specific responses to ETL signaling have been reported in several plant systems, where differences in hormone sensitivity and regulatory networks can lead to distinct phytochemical accumulation patterns [50]. Such regulatory variation may influence the activation of phenylpropanoid-associated pathways [45] and contribute to the stronger isoflavone response observed in ML compared with SL. Changes in composition, expression patterns, and promoter responsiveness of transcription factor complexes, such as MYB, may result in differential activation of phenylpropanoid-associated genes under the same hormonal stimulation [51, 52]. Therefore, differences in transcriptional regulation and post-synthesis transformation capacity may contribute to the distinct ETL responses observed between species [1, 18].

ETL has been reported to participate in plant defense responses and antioxidant activity, partly through the regulation of phenolic and flavonoid biosynthesis. In this study, ETL treatment significantly increased the TF content of ML, which was consistent with the enhanced antioxidant activity as indicated by decreased IC₅₀ values ​​in DPPH and ABTS radical scavenging assays (Supplementary Fig. 2). These results are consistent with previous reports showing that ETL or ethephon treatment increases the expression of genes such as *CHS* and *CHI*, rate-limiting enzymes in the flavonoid biosynthetic pathway, in various species, including blueberry, soybean, and mung bean [1, 53]. At the signal transduction level, ethylene perception and signal transduction have been extensively studied in model plants. Activation of *EIN3* and *ERF* family members has been shown to regulate stress-responsive gene expression and, in certain plant systems, to influence phenylpropanoid and flavonoid-related pathways [12, 54].

The observed increased flavonoid accumulation in this study is consistent with this established regulatory mechanism. However, future studies integrating transcriptomic and enzyme analyses are needed to clarify whether canonical ETL signaling components directly mediate the metabolic changes observed under these experimental conditions. The increased expression of biosynthetic genes is associated with increased enzyme activity and enhanced metabolic flux toward flavonoid intermediates and end products [55]. Because flavonoids possess potent free radical scavenging and metal chelating properties, their accumulation may contribute to enhanced antioxidant capacity. Furthermore, flavonoids are known to inhibit digestive enzymes such as α-glucosidase and pancreatic lipase through interactions at the catalytic site [1, 2, 18], which may partially explain the increased enzyme inhibitory activity observed in ML after ETL treatment. The more pronounced response in ML compared to SL may reflect species-specific differences in the regulation of flavonoid biosynthesis, possibly related to variations in transcription factor networks such as the MYB complex [2, 8, 51]. Further molecular studies are needed to elucidate the regulatory mechanisms underlying these differential responses.

DNA is particularly susceptible to oxidative damage caused by ROS, which can cause strand breaks and structural conversion from SC to open circular DNA (OC) or linear DNA (LIN) forms [56]. In this study, the most prominent DNA-protective effect was detected in ML extracts after ETL treatment, suggesting that the accumulation of isoflavone, flavonoid, and phenolic compounds may contribute to the observed *in vitro* DNA protection under oxidative stress conditions [57]. ETL has been reported to induce the accumulation of flavonols such as quercetin and kaempferol in guard cells, resulting in decreased intracellular ROS levels and increased protection against oxidative stress [1]. In particular, ETL-induced isoflavones, including DAE, have been demonstrated to inhibit DNA damage through iron chelation and free radical scavenging mechanisms [2, 58]. Moreover, ETL activates the phenylpropanoid pathway and modulates antioxidant metabolism through ERF-type transcription factors, thereby improving flavonoid and polyphenol accumulation and ROS resistance [59]. The observation that DNA protection was extended to mung bean stems and roots after ETL treatment supports the hypothesis that ETL triggers a systemic metabolic response [1].

Consistently, previous research has reported strong antioxidant and DNA-protective activities associated with increased isoflavone levels in mung beans, with a positive correlation detected between ETL-induced isoflavone accumulation and improved DNA integrity [1]. Moreover, the superior SC band retention in ETL-treated mung beans across all concentrations in our study supports these mechanisms and suggests that ETL-associated metabolite modulation may have potential relevance for metabolite-oriented crop management strategies; however, further validation under physiological and agronomic conditions is required.

## Conclusions

ETL treatment was associated with coordinated changes in both primary and secondary-metabolite profiles in soybean and mung bean plants cultivated in a vertical farming system.

ETL exposure increased antioxidant-related indices, including TP and TF contents, enhanced isoflavone accumulation, altered FAA and FA compositions, and was associated with improved *in vitro* bioactivities, including antioxidant capacity, digestive enzyme inhibition, and plasmid DNA protection. These metabolic changes occurred alongside a measurable reduction in biomass, indicating that ETL treatment influenced both growth and metabolite profiles under the tested condition; however, causal relationships between biomass reduction and metabolite enrichment were not directly established. Comparative analysis further revealed species-dependent differences at the metabolite level. Mung bean exhibited greater increases in isoflavones and phenolic-related antioxidant activity, whereas soybean maintained relatively higher levels of selected essential amino acids. Because gene expression, enzyme activity, and ethylene signaling components were not directly analyzed, the observed metabolite shifts should be interpreted as metabolite-level responses rather than confirmed regulation of metabolic pathways. In addition, since only a single ETL concentration and treatment regime were applied, the results represent a proof-of-concept under specific experimental conditions rather than an optimized or broadly applicable treatment strategy. Overall, the present findings provide metabolite-level evidence that ETL treatment can modulate functional metabolite accumulation in legumes under controlled cultivation systems, while highlighting the need for further molecular and agronomic validation.

## Supplementary Information

Supplementary Material 1: Supplementary Fig. 1. Representative photographs of soybean and mung bean plants and leaves under control and ethylene treatment conditions. (A) Untreated soybean plants and leaves (SL-CTL); (B) ethylene-treated soybean plants and leaves (SL-ETL); (C) untreated mung bean plants and leaves (ML-CTL); and (D) ethylene-treated mung bean plants and leaves (ML-ETL).

Supplementary Material 2: Supplementary Fig. 2. Total isoflavone accumulation per leaf under ethylene treatments. Values are expressed as mean ± SD (n = 5). Different lowercase letters indicate significant differences among treatments within each species, as determined by one-way ANOVA followed by Tukey’s multiple comparison test (p < 0.05).

Supplementary Material 3: Supplementary Fig. 3. Pearson correlation analysis between total isoflavone contents and bioactivity indices. Correlation coefficients were calculated between total isoflavone contents and functional parameters, including DPPH and ABTS radical scavenging activities, α-glucosidase inhibition, and pancreatic lipase inhibition. Circle size and color intensity represent the strength and direction of correlation (red, positive; blue, negative). Asterisks indicate statistically significant correlations (*p < 0.05, **p < 0.01, ***p < 0.001).

Supplementary Material 4: Supplementary Fig. 4. Pearson correlation analysis among different metabolite classes, including isoflavones, amino acids, and fatty acids. (A) Pearson correlation heatmap showing relationships between isoflavone derivatives and amino acids. (B) Pearson correlation heatmap showing the relationships between isoflavone derivatives and fatty acids. Circle size and color intensity represent the strength and direction of the correlation (red, positive; blue, negative), and asterisks indicate statistically significant correlations (*p < 0.05, **p < 0.01, ***p < 0.001).

Supplementary Material 5: Table S1. Comparison of fatty acid contents of soybean and mung bean leaves following ethylene treatment.

Supplementary Material 6: Table S2. Comparison of free amino acid contents of soybean and mung bean leaves following ethylene treatments.

Supplementary Material 7: Table S3. Comparison of isoflavone and flavone contents for 50% MeOH extracts of soybean and mung bean leaves treated with ethylene.

## Data Availability

The datasets generated and/or analyzed during the current study are included in this published article and its supplementary information files. Additional data are available from the corresponding author on reasonable request.

## Abbreviations

ETL: Ethylene
ML: Mung bean leaves
SL: Soybean leaves
FA: Fatty acid
FAA: Free amino acid
GABA: γ-aminobutyric acid
DPPH: 2,2-diphenyl-1-pyridylhydrazine
ABTS: 2,2′-azino-bis(3-ethylbenzothiazolin-6-sulfonic acid)
TF: Total flavonoid
TP: Total phenolic
CHS: Chalcone synthase
IFS: Isoflavone synthase
CHI: Chalcone isomerase
