# Supplementary material for: Ethylene-driven enhancement of bioactive metabolites and in vitro functionality in soybean (Glycine max (L.) Merr.) and mung bean (Vigna radiata (L.) Wilczek) leaves grown in vertical farms: a comparative study
Source: BMC Plant Biol. 2026 Apr 30;26:1042. doi: 10.1186/s12870-026-08829-8 (PMC13274195; doi:10.1186/s12870-026-08829-8)
Supplement: Supplementary file 1 — Supplementary Material 1: Supplementary Fig. 1. Representative photographs of soybean and mung bean plants and leaves under control and ethylene treatment conditions. (A) Untreated soybean plants and leaves (SL-CTL); (B) ethylene-treated soybean plants and leaves (SL-ETL); (C) untreated mung bean plants and leaves (ML-CTL); and (D) ethylene-treated mung bean plants and leaves (ML-ETL). [file 12870_2026_8829_MOESM1_ESM.docx]

**Supplementary Information**

**Supplementary Figure 1**

**
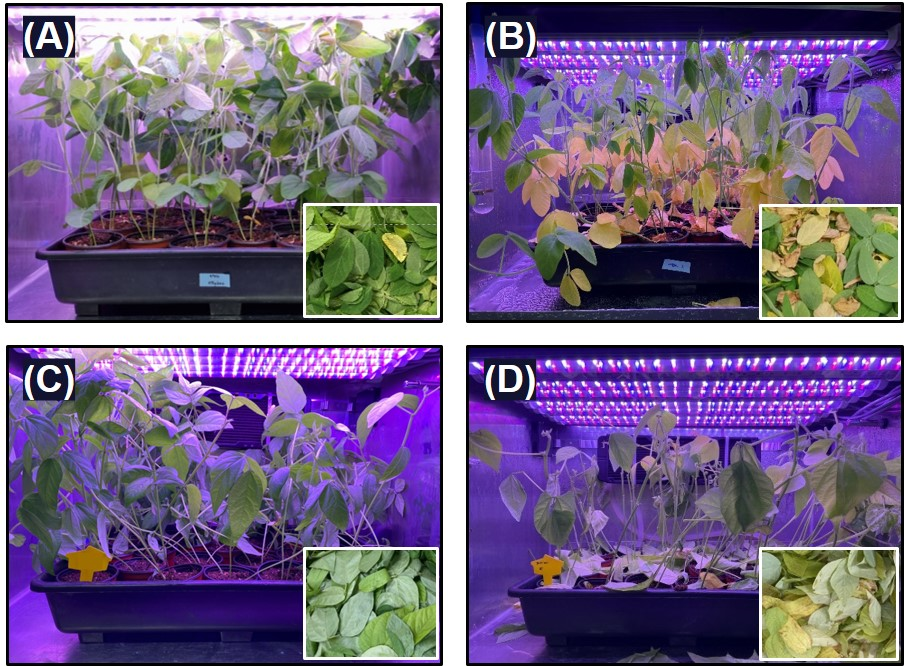
**

**Supplementary Fig. 1.** Representative photographs of soybean and mung bean plants and leaves under control and ethylene treatment conditions. (A) Untreated soybean plants and leaves (SL-CTL); (B) ethylene-treated soybean plants and leaves (SL-ETL); (C) untreated mung bean plants and leaves (ML-CTL); and (D) ethylene-treated mung bean plants and leaves (ML-ETL).
