# Supplementary material for: Ethylene-driven enhancement of bioactive metabolites and in vitro functionality in soybean (Glycine max (L.) Merr.) and mung bean (Vigna radiata (L.) Wilczek) leaves grown in vertical farms: a comparative study
Source: BMC Plant Biol. 2026 Apr 30;26:1042. doi: 10.1186/s12870-026-08829-8 (PMC13274195; doi:10.1186/s12870-026-08829-8)
Supplement: Supplementary file 2 — Supplementary Material 2: Supplementary Fig. 2. Total isoflavone accumulation per leaf under ethylene treatments. Values are expressed as mean ± SD (n = 5). Different lowercase letters indicate significant differences among treatments within each species, as determined by one-way ANOVA followed by Tukey’s multiple comparison test (p < 0.05). [file 12870_2026_8829_MOESM2_ESM.docx]

**Supplementary Information**

**Supplementary Figure 2**

**Supplementary Fig. 2.** Total isoflavone accumulation per leaf under ethylene treatments. Values are expressed as mean ± SD (*n* = 5). Different lowercase letters indicate significant differences among treatments within each species, as determined by one-way ANOVA followed by Tukey’s multiple comparison test (*p* < 0.05).
