# Supplementary material for: Ethylene-driven enhancement of bioactive metabolites and in vitro functionality in soybean (Glycine max (L.) Merr.) and mung bean (Vigna radiata (L.) Wilczek) leaves grown in vertical farms: a comparative study
Source: BMC Plant Biol. 2026 Apr 30;26:1042. doi: 10.1186/s12870-026-08829-8 (PMC13274195; doi:10.1186/s12870-026-08829-8)
Supplement: Supplementary file 3 — Supplementary Material 3: Supplementary Fig. 3. Pearson correlation analysis between total isoflavone contents and bioactivity indices. Correlation coefficients were calculated between total isoflavone contents and functional parameters, including DPPH and ABTS radical scavenging activities, α-glucosidase inhibition, and pancreatic lipase inhibition. Circle size and color intensity represent the strength and direction of correlation (red, positive; blue, negative). Asterisks indicate statistically significant correlations (*p < 0.05, **p < 0.01, ***p < 0.001). [file 12870_2026_8829_MOESM3_ESM.docx]

**Supplementary Information**

**Supplementary Figure 3**

**
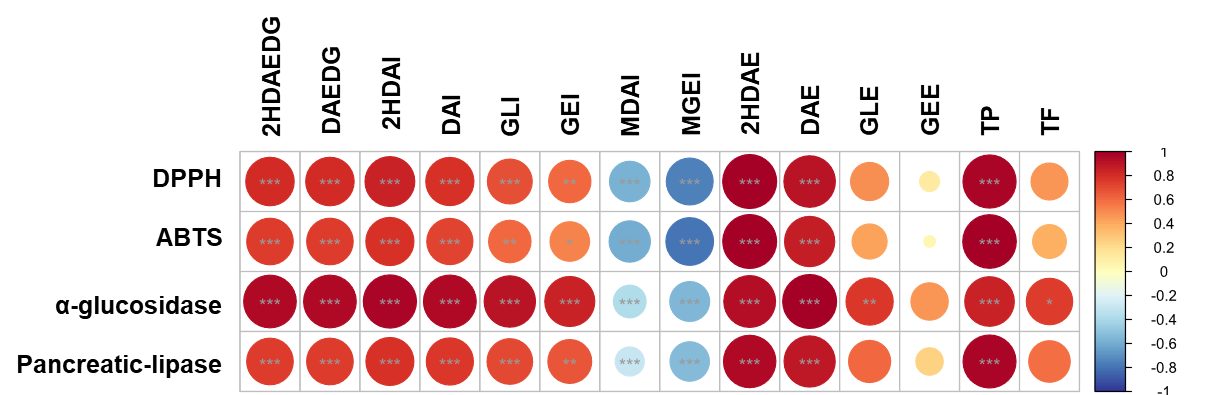
**

**Supplementary Fig. 3.** Pearson correlation analysis between total isoflavone contents and bioactivity indices. Correlation coefficients were calculated between total isoflavone contents and functional parameters, including DPPH and ABTS radical scavenging activities, α-glucosidase inhibition, and pancreatic lipase inhibition. Circle size and color intensity represent the strength and direction of correlation (red, positive; blue, negative). Asterisks indicate statistically significant correlations (**p* < 0.05, ***p* < 0.01, ****p* < 0.001).
