# Supplementary material for: Ethylene-driven enhancement of bioactive metabolites and in vitro functionality in soybean (Glycine max (L.) Merr.) and mung bean (Vigna radiata (L.) Wilczek) leaves grown in vertical farms: a comparative study
Source: BMC Plant Biol. 2026 Apr 30;26:1042. doi: 10.1186/s12870-026-08829-8 (PMC13274195; doi:10.1186/s12870-026-08829-8)
Supplement: Supplementary file 4 — Supplementary Material 4: Supplementary Fig. 4. Pearson correlation analysis among different metabolite classes, including isoflavones, amino acids, and fatty acids. (A) Pearson correlation heatmap showing relationships between isoflavone derivatives and amino acids. (B) Pearson correlation heatmap showing the relationships between isoflavone derivatives and fatty acids. Circle size and color intensity represent the strength and direction of the correlation (red, positive; blue, negative), and asterisks indicate statistically significant correlations (*p < 0.05, **p < 0.01, ***p < 0.001). [file 12870_2026_8829_MOESM4_ESM.docx]

**Supplementary Information**

**Supplementary Figure 4**

**
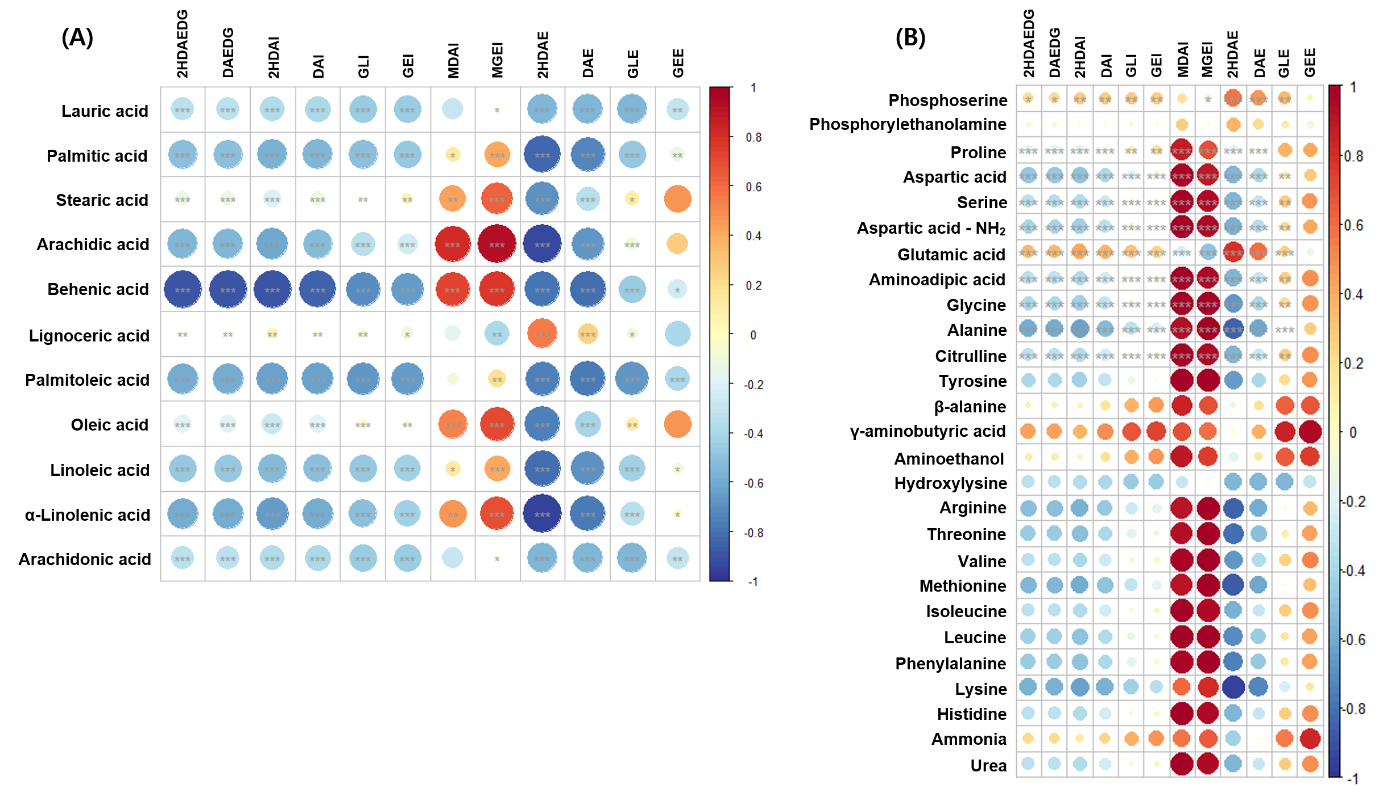
**

**Supplementary Fig. 4.** Pearson correlation analysis among different metabolite classes, including isoflavones, amino acids, and fatty acids. (A) Pearson correlation heatmap showing relationships between isoflavone derivatives and amino acids. (B) Pearson correlation heatmap showing the relationships between isoflavone derivatives and fatty acids. Circle size and color intensity represent the strength and direction of the correlation (red, positive; blue, negative), and asterisks indicate statistically significant correlations (**p* < 0.05, ***p* < 0.01, ****p* < 0.001).
