# Supplementary material for: Ethylene-driven enhancement of bioactive metabolites and in vitro functionality in soybean (Glycine max (L.) Merr.) and mung bean (Vigna radiata (L.) Wilczek) leaves grown in vertical farms: a comparative study
Source: BMC Plant Biol. 2026 Apr 30;26:1042. doi: 10.1186/s12870-026-08829-8 (PMC13274195; doi:10.1186/s12870-026-08829-8)
Supplement: Supplementary file 5 — Supplementary Material 5: Table S1. Comparison of fatty acid contents of soybean and mung bean leaves following ethylene treatment. [file 12870_2026_8829_MOESM5_ESM.docx]

**Table S1.** Comparison of fatty acid contents of soybean and mung bean leaves following ethylene treatment.

| **Contents^1)^**  **(mg/100 g)** | **Treatment of ethylene^2)^** | | | |
| --- | --- | --- | --- | --- |
|  | **SL-CTL** | **SL-ETL** | **ML-CTL** | **ML-ETL** |
| **Saturated fatty acids** |  |  |  |  |
| Lauric acid(C12:0) | 8.7 ± 0.33^a^ | nd^3)^ | nd | nd |
| Palmitic acid (C16:0) | 273.7 ± 8.11^a^ | 244.7 ± 5.96^b^ | 224.5 ± 6.97^c^ | 223.3 ± 3.16^c^ |
| Stearic acid (C18:0) | 76.8 ± 2.11^a^ | 73.6 ± 2.05^a^ | 54.8 ± 1.33^c^ | 66.0 ± 1.46^b^ |
| Arachidic acid (C20:0) | 9.4 ± 0.33^b^ | 13.9 ± 0.40^a^ | nd | nd |
| Behenic acid (C22:0) | 10.1 ± 0.27^c^ | 18.3 ± 0.50^a^ | 11.9 ± 0.16^b^ | nd |
| Lignoceric acid (C24:0) | 13.2 ± 0.34^c^ | 15.4 ± 0.45^b^ | 18.9 ± 0.64^a^ | 15.8 ± 0.28^b^ |
| Total | 391.9 | 365.9 | 310.1 | 305.1 |
| **Unsaturated fatty acids** |  |  |  |  |
| Palmitoleic acid (C16:1) | 48.6 ± 1.29^a^ | 14.4 ± .0.28^b^ | 10.9 ± 0.3^c^ | nd |
| Elaidic acid (C18:1t) | nd | nd | nd | nd |
| Oleic acid (C18:1c) | 48.3 ± 1.32^a^ | 47.3 ± 1.86^a^ | 27.5 ± 0.62^c^ | 37.2 ± 0.93^b^ |
| Linoleic acid (C18:2c) | 215.5 ± 4.91^a^ | 127.1 ± 4.23^b^ | 61.4 ± 2.23^d^ | 66.6 ± 1.52^c^ |
| α-Linolenic acid (C18:3n3) | 495.6 ± 11.90^a^ | 440.5 ± 15.94^b^ | 263.5 ± 8.11^c^ | 252.5 ± 5.45^d^ |
| Eicosadienoic acid (C20:2) | nd | nd | nd | nd |
| Arachidonic acid(C20:4n6) | 8.6 ± 0.27^a^ | nd | nd | nd |
| Total | 816.6 | 629.3 | 363.3 | 356.3 |
| **Sum of fatty acids** | **1,208.5** | **995.2** | **673.4** | **661.4** |
| ^1)^All values are expressed as the mean ± SD of pentaplicate determination. Different small letters (a–d) correspond to significant differences related to the same row, as determined by the ANOVA and followed by Tukey's multiple tests (*p* < 0.05).  ^2)^Treatment conditions in plant chamber: light intensity 143.20 μmol/m^-2^/s^-1^ (16 h photoperiod), temperature 25 ^º^C±5, humidity 90%±5, and ethylene concentration 10,000 ppm applied for 24 h, repeated twice (total exposure time: 48 h). Abbreviations: SL-CTL, control soybean leaves (untreated); SL-ETL, ethylene-treated soybean leaves; ML-CTL, control mung bean leaves (untreated); ML-ETL, ethylene-treated mung bean leaves.  ^3)^nd: not detected. | | | | |
