# Supplementary material for: Ethylene-driven enhancement of bioactive metabolites and in vitro functionality in soybean (Glycine max (L.) Merr.) and mung bean (Vigna radiata (L.) Wilczek) leaves grown in vertical farms: a comparative study
Source: BMC Plant Biol. 2026 Apr 30;26:1042. doi: 10.1186/s12870-026-08829-8 (PMC13274195; doi:10.1186/s12870-026-08829-8)
Supplement: Supplementary file 6 — Supplementary Material 6: Table S2. Comparison of free amino acid contents of soybean and mung bean leaves following ethylene treatments. [file 12870_2026_8829_MOESM6_ESM.docx]

**Table S2.** Comparison of free amino acid contents of soybean and mung bean leaves following ethylene treatments.

| **Contents^1)^ (mg/100 g)** | **Treatment of ethylene^2)^** | | | |
| --- | --- | --- | --- | --- |
|  | **SL-CTL** | **SL-ETL** | **ML-CTL** | **ML-ETL** |
| **Non-essential amino acids** |  |  |  |  |
| Phosphoserine | 15.85 ± 0.44^c^ | 28.10 ± 0.32^b^ | 31.39 ± 0.62^a^ | 28.04 ± 0.81^b^ |
| Taurine | nd^3)^ | nd | nd | nd |
| Phosphorylethanolamine | 1.66 ± 0.04^d^ | 6.40 ± 0.19^b^ | 7.76 ± 0.17^a^ | 4.95 ± 0.07^c^ |
| Proline | 8.42 ± 0.14^c^ | 45.21 ± 0.24^a^ | 25.05 ± 0.32^b^ | 21.15 ± 0.68^b^ |
| Aspartic acid | 9.44 ± 0.34^c^ | 34.52 ± 1.05^a^ | 14.49 ± 0.24^b^ | 7.74 ± 0.21^c^ |
| Serine | 13.85 ± 0.41^b^ | 42.89 ± 0.47^a^ | 14.42 ± 0.42^b^ | 13.80 ± 0.47^b^ |
| Aspartic acid - NH_2_ | 23.46 ± 0.83^c^ | 189.04 ± 6.25^a^ | 30.79 ± 0.81^b^ | 14.13 ± 0.48^d^ |
| Glutamic acid | 13.84 ± 0.37^d^ | 38.84 ± 0.88^c^ | 71.29 ± 2.39^a^ | 58.69 ± 1.23^b^ |
| Aminoadipic acid | nd | 10.93 ± 0.35^a^ | nd | nd |
| Glycine | 7.57 ±0.17 ^b^ | 9.33 ± 0.25^a^ | 7.12 ± 0.13^b^ | 7.21 ± 0.12^b^ |
| Alanine | 36.70 ± 0.97^b^ | 63.76 ± 1.96^a^ | 22.17 ± 0.51^c^ | 15.96 ± 0.21^d^ |
| Citrulline | nd | 2.68 ± 0.06^a^ | nd | nd |
| Tyrosine | 14.27 ± 0.41^b^ | 73.14 ± 1.69^a^ | 3.81 ± 0.04^c^ | 4.43 ± 0.07^c^ |
| β-alanine | 1.01 ± 0.02^d^ | 4.61 ± 0.12^a^ | 1.97 ± 0.03^c^ | 2.80 ± 0.10^b^ |
| γ-aminobutyric acid | 118.68 ± 3.31^c^ | 192.33 ± 6.22^a^ | 100.64 ± 1.93^d^ | 174.07 ± 5.6^b^ |
| Aminoethanol | 18.33 ± 0.38^c^ | 23.42 ± 0.76^a^ | 18.85 ± 0.35^c^ | 20.67 ± 0.56^b^ |
| Hydroxylysine | 10.41 ± 0.20^a^ | nd | nd | nd |
| Arginine | 16.32 ± 0.50^b^ | 34.40 ± 1.23^a^ | nd | nd |
| Total | 309.81 | 814.14 | 349.75 | 373.64 |
| **Essential amino acids** |  |  |  |  |
| Threonine | 14.51 ± 0.53^b^ | 25.82 ± 0.54^a^ | 6.05 ± 0.20^c^ | 7.39 ± 0.18^c^ |
| Valine | 37.94 ± 1.04^b^ | 81.50 ± 2.01^a^ | 21.06 ± 0.70^d^ | 28.82 ± 1.09^c^ |
| Methionine | 2.17 ± 0.04^b^ | 3.82 ± 0.14^a^ | 0.74 ± 0.02^c^ | 0.62 ± 0.01^d^ |
| Isoleucine | 12.18 ± 0.25^b^ | 70.98 ± 2.04^a^ | 9.61 ± 0.26^d^ | 11.20 ± 0.4^c^ |
| Leucine | 27.96 ± 0.49^b^ | 102.96 ± 3.41^a^ | 6.52 ± 0.13^c^ | 6.08 ± 0.17^c^ |
| Phenylalanine | 16.67 ± 0.18^b^ | 60.62 ± 1.75^a^ | nd | nd |
| Lysine | 24.83 ± 0.74^a^ | 24.44 ± 0.67^a^ | 3.32 ± 0.07^b^ | 3.42 ± 0.06^b^ |
| Histidine | nd | 16.92 ± 0.36^a^ | nd | nd |
| Total | 136.26 | 387.06 | 47.30 | 57.53 |
| **Sum of amino acids** | **446.07** | **1,201.20** | **397.05** | **431.17** |
| **Urea metabolism** |  |  |  |  |
| Ammonia | 10.41 ± 0.30^b^ | 13.25 ± 0.23^a^ | nd | 10.29 ±0.22^b^ |
| Urea | nd | 14.54 ± 0.40 | nd | nd |
| Total | 10.41 | 27.79 | nd | 10.29 |
| ^1)^All values are expressed as the mean ± SD of pentaplicate determination. Different small letters (a–d) correspond to significant differences related to the same row, as determined by the ANOVA and followed by Tukey's multiple tests (*p* < 0.05).  ^2)^ Treatment conditions in plant chamber: light intensity 143.20 μmol/m^-2^/s^-1^ (16 h photoperiod), temperature 25 ^º^C±5, humidity 90%±5, and ethylene concentration 10,000 ppm applied for 24 h, repeated twice (total exposure time: 48 h). Abbreviations: SL-CTL, control soybean leaves (untreated); SL-ETL, ethylene-treated soybean leaves; ML-CTL, control mung bean leaves (untreated); and ML-ETL, ethylene-treated mung bean leaves.  ^3)^ nd: not detected. | | | | |
