# Supplementary material for: Ethylene-driven enhancement of bioactive metabolites and in vitro functionality in soybean (Glycine max (L.) Merr.) and mung bean (Vigna radiata (L.) Wilczek) leaves grown in vertical farms: a comparative study
Source: BMC Plant Biol. 2026 Apr 30;26:1042. doi: 10.1186/s12870-026-08829-8 (PMC13274195; doi:10.1186/s12870-026-08829-8)
Supplement: Supplementary file 7 — Supplementary Material 7: Table S3. Comparison of isoflavone and flavone contents for 50% MeOH extracts of soybean and mung bean leaves treated with ethylene. [file 12870_2026_8829_MOESM7_ESM.docx]

| **Table S3. Comparison of isoflavone and flavone contents for 50% MeOH extracts of soybean and mung bean leaves treated with ethylene** | | | | | | | | | | | | | | |
| --- | --- | --- | --- | --- | --- | --- | --- | --- | --- | --- | --- | --- | --- | --- |
| **Contents^1)^**  **(µg/g)** | | **Organs of the soybean and mung bean leaves** | | | | | | | | | | | | |
|  |  | **SL-CTL** | | | | **SL-ETL** | | | **ML-CTL** | | | | **ML-ETL** | |
| **Diglycosides** | |  |  | | | | | | |  | | | | |
| 2HDAEDG | | nd^2)^ | | | nd | | | nd | | | | 1,169.87 ± 53.19^a^ | | |
| DAEDG | | nd | | | nd | | | nd | | | | 1,839.23 ± 25.75^a^ | | |
| Total | | nd | | | nd | | | nd | | | | 3,009.09 | | |
| **Malonyl Glycosides** | | | | | | | | | | | | | | |
| MDAI | | 112.16 ± 2.48^b^ | | | 2,194.02 ± 7.62^a^ | | | nd | | | | nd | | |
| MGEI | | 866.87 ± 2.70^b^ | | | 2,661.02 ± **1**2.28^a^ | | | nd | | | | nd | | |
| Total | | 979.03 | | | 4,860.04 | | | nd | | | | nd | | |
| **Glycosides** | |  | | | | | | | | | | | | |
| 2HDAI | | nd | | | nd | | | 1,009.58 ± 56.53^b^ | | | | 12,118.25 ± 53.24^a^ | | |
| DAI | | 102.85 ± 2.60^d^ | | | 2,423.77 ± 11.87^b^ | | | 918.78 ± 55.01^c^ | | | | 22,828.21 ± 20.03^a^ | | |
| GLI | | nd | | | 156.24 ± 0.90^b^ | | | nd | | | | 533.75 ± 5.89^a^ | | |
| GEI | | 288.31 ± 11.02^c^ | | | 1,112.59 ± 8.22^b^ | | | 178.17 ± 3.25^d^ | | | | 2,502.84 ± 8.30^a^ | | |
| Total | | 391.16 | | | 3,536.36 | | | 2,106.53 | | | | 37,983.05 | | |
| **Aglycones** | |  | | | | | | | | | | | | |
| 2HDAE | | nd | | | nd | | | 311.42 ± 43.26^b^ | | | | 483.03 ± 7.45^a^ | | |
| DAE | | nd | | | 155.80 ± 1.54^c^ | | | 260.07 ± 32.50^b^ | | | | 889.24 ± 15.67^a^ | | |
| GLE | | nd | | | 108.38 ± 2.98^b^ | | | nd | | | | 179.91 ± 9.67^a^ | | |
| GEE | | 70.24 ± 1.55^c^ | | | 150.94 ± 0.33^b^ | | | 19.68 ± 2.35^d^ | | | | 164.32 ± 4.62^a^ | | |
| Total | | 70.24 | | | 306.74 | | | 591.18 | | | | 1,716.49 | | |
| **Total isoflavones** | | **1,440.43** | | | **8,703.14** | | | **2,697.71** | | | | **42,708.64** | | |
| *Abbreviations: SL-CTL, control soybean leaves (untreated); SL-ETL, ethylene-treated soybean leaves; ML-CTL, control mung bean leaves (untreated); and ML-ETL, ethylene-treated mung bean leaves; 2HDAEDG, 2'-hydroxydaidzein-4',7-*O*-diglucoside; DAEDG, daidzein-4',7-*O*-diglucoside; MDAI, malonyldaidzin; MGEI, malonylgenistin; 2HDAI, 2'-hydroxydaidzin; DAI, daidzin; GLI, glycitin; GEI, genistin; 2HDAE, 2'-hydroxydaidzein; DAE, daidzein; GLE, glycitein; GEE, genistein.  ^1)^ All values are expressed as the mean ± SD of pentaplicate determination. Different small letters (a–d) correspond to significant differences related to the same row, as determined by the ANOVA and followed by Tukey's multiple tests (*p* < 0.05).  ^2)^ nd: not detected. | | | | | | | | | | | | | | |
